# Peak cement‐related CO_2_ emissions and the changes in drivers in China

**DOI:** 10.1111/jiec.12839

**Published:** 2019-02-08

**Authors:** Yuli Shan, Ya Zhou, Jing Meng, Zhifu Mi, Jingru Liu, Dabo Guan

**Affiliations:** 1 Tyndall Centre of Climate Change Research and School of Environmental Science, University of East Anglia, Norwich, UK; 2 School of Management, Guangdong University of Technology, Guangzhou, China; 3 Big Data Strategic Research Institute, Guangdong University of Technology, Guangzhou, China; 4 Department of Politics and International Studies, University of Cambridge, Cambridge, UK; 5 Bartlett School of Construction and Project Management, University College London, London, UK; 6 State Key Laboratory of Urban and Regional Ecology, Research Centre for Eco‐Environmental Sciences, Chinese Academy of Sciences, Beijing, China; 7 Department of Earth System Sciences, Tsinghua University, Beijing, China; 8 Water Security Research Centre, School of International Development, University of East Anglia, NR4 7TJ Norwich, UK

**Keywords:** cement industry, China, CO_2_ emissions, driving forces, index decomposition analysis, industrial ecology

## Abstract

In order to fight against the climate change, China has set a series of emission reduction policies for super‐emitting sectors. The cement industry is the major source of process‐related emissions, and more attention should be paid to this industry. This study calculates the process‐related, direct fossil fuel–related, and indirect electricity‐related emissions from China’s cement industry. The study finds that China’s cement‐related emissions peaked in 2014. The emissions are, for the first time, divided into seven parts based on the cement used in different new building types. The provincial emission analysis finds that developed provinces outsourced their cement capacities to less developed regions. This study then employs index decomposition analysis to explore the drivers of changes in China’s cement‐related emissions. The results show that economic growth was the primary driver of emission growth, while emission intensity and efficiency were two offsetting factors. The changes in the construction industry’s structure and improvement in efficiency were the two major drivers that contributed to the decreased emissions since 2014.

**Supplementary Information:**

The online version of this article (doi:10.1111/jiec.12839) contains supplementary material, which is available to authorized users.

## INTRODUCTION

Being the largest CO_2_ emitter and energy consumer (Guan, Peters, Weber, & Hubacek, 2009), China is taking increasing responsibility for global climate actions. To reduce greenhouse gas (GHG) emissions in more effective and efficient ways, China has set a series of relevant strategies and specific policies targeting super‐emitting sectors (NDRC, 2009), including power generation (NDRC, 2009), coal mining (NDRC, 2009), and the iron and steel industry (MIIT, 2009). Cement is one of the largest key sources of process‐related emissions in China and worldwide. According to China’s official GHG emission inventories from National Communications on Climate Change (as shown in Figure S1), process‐related GHG emissions from the cement lime industry reached 157.78 million tons (Mt) (or 57% of the total process‐related emissions) in 1994 (NDRC, 2004), 411.57 Mt (or 72%) in 2005 (NDRC, 2013), and 834.03 Mt (or 70%) in 2012 (NDRC, 2009). With such a large amount and rapid growth of GHG emissions, the cement industry has become a key sector in GHG emissions mitigation. Indeed, several policies have been proposed to reduce the energy consumption and emission intensity of the cement industry in China. In the latest 13th five‐year plan for the cement industry, the government aims to achieve a 30% reduction of the air pollutant emissions of the cement industry in 2020 compared to the 2015 level (China Cement Association [CCA], 2009). The energy consumption per tonne of clinker production should be kept under 105 kg coal equivalent in 2020, while those of 2015 amounted to 112 kg. With the efforts of the government, the process‐related CO_2_ emissions from China’s cement industry peaked in 2014 (Shan, Guan, Zheng, et al., 2018), maintaining the same trend as China’s total emissions (Guan et al., 2018).

Accurate accounts of the cement‐related emissions and understanding the driving forces of the changes in emissions is of considerable value, and especially has practical significance for further emission reduction policymaking. Oda, Maksyutov, and Andres (2018) developed a global high spatial resolution gridded CO_2_ emissions data inventory including cement production as a part of nonpoint emission sources. Şanal (2018) evaluated the CO_2_ emissions of different types of cement and discussed emission reduction capacities of cement replacement in concrete production. Andrew (2018) presented a new analysis of global process emissions from cement production, which were 30 % lower than those reported by the Global Carbon Project (Le Quéré et al., 2015). Apart from the carbon source function, Xi et al. (2016) found that carbonation of cement materials has offset 43% of CO_2_ emissions from cement production from 1930 to 2013.

Previous studies have employed the index decomposition analysis (IDA)–logarithmic mean Divisia index (LMDI) model to analyze the variations of carbon emissions. Branger and Quirion (2015) investigated the changes of CO_2_ emissions in the European cement industry from 1990 to 2012 and found that most of the emission change (activity, clinker trade, thermal and electrical energy efficiency, and electricity decarbonization) could be attributed to the activity effect. When it comes to China, Xu, Fleiter, Eichhammer, and Fan (2012) got a similar result. They found that the activity effect (calcining process and electricity consumption) was the main driver of cement‐related emissions increase while clinker share, structural shift, and kiln efficiency were main negative drivers. Wang, Zhu, and Geng (2013) identified the main drivers that influence China’s cement‐related GHG emissions. Lin and Zhang (2016) found that the labor productivity was the major driving force to increase the cement‐related CO_2_ emissions from 1991 to 2010.

Despite that nearly all previous studies in emission accounts and analysis involved the cement process in the emission inventories, the emissions are usually calculated with the cement production (Liu et al., 2015; Shan, Guan, Hubacek, et al., 2018; Shan, Liu, Liu, et al., 2016). As the process‐related CO_2_ emissions are majorly produced alongside the clinker production, such a cement production‐based calculation method is not accurate enough. It may overlook the regional diversity in the cement manufacturing technique and cement–clinker ratio. Therefore, some recent studies calculated the emissions with clinker’s production (Cai, Wang, He, & Geng, 2016).

Considering the regional diversity in China’s cement manufacturing, this study first investigates the emissions from China and its regions’ cement industry. This study examines the process‐related CO_2_ emissions (calculated based on the clinker productions), direct emissions from fossil fuel combustion, and indirect emissions induced by purchased electricity in the cement industry. The cement‐related CO_2_ emissions are divided into seven parts according to the cement demands of different new building types for the first time. This study also describes the regional diversity in cement production and the cement production capacity shifts among the provinces.

This study then employs the LMDI method to break down the changes in China’s cement‐related CO_2_ emissions into four drivers, including the construction industry’s structure, emission intensity, efficiency, and economic growth. To the best of our knowledge, our study is the first to examine the factor of “construction industry’s structure,” which is measured by the cement used for different new building types, in the analysis of the drivers of China’s cement‐related emissions. We particularly compared the changes in drivers before and after these emissions peaked in 2014.

Our study provides robust and transparent data support for further environmental evaluations and emission reduction/sustainable production policymaking for the cement industry in China.^1^

## METHODS AND DATA SOURCES

### 2.1 Cement production process and related emissions

**Figure 1:**
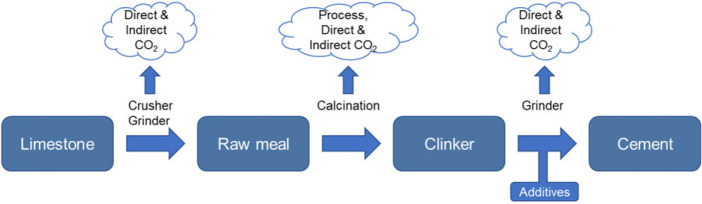
Cement production process and emission calculation

Cement is normally produced in three steps, as shown in Figure 1 (Worrell, Price, Martin, Hendriks, & Meida, 2001). First, the limestone (primarily CaCO_3_) is crushed and ground into raw meal. Then, the raw meal is calcined in kilns to clinker. Finally, the clinker is ground together with additives (fly ash, pozzolana, gypsum, anhydrite, etc.) to form cement.

The crusher and grinder are physical reactions that do not emit any CO_2_ during the process. In contrast, calcination is a chemical reaction, during which the process‐related CO_2_ may be emitted. The process‐related emissions are the CO_2_ emitted as a result of chemical reactions in the production process rather than the energy combusted by industry (Shan, Liu, & Guan, 2016). During the calcination of raw meal, the limestone is heated to lime (CaO) and CO_2_; see Equation 1. The CO_2_ emissions are process‐related emissions during the cement production process.

1 $$ \mathrm{CaC}\ {\mathrm{O}}_3\genfrac{}{}{0pt}{}{\Delta}{=}\ \mathrm{CaO}+\mathrm{C}{\mathrm{O}}_2\uparrow . $$

Although the crushing and grinding processes do not emit process‐related emissions, they consume abundant energy for power, such as coal and electricity. The CO_2_ emitted during coal combustion and electricity generation is counted as direct emissions and indirect emissions, respectively (Liu, 2016b). The indirect emissions are normally produced in power plants rather than the cement plants.

Overall, this study considers the process‐related CO_2_ emissions, direct CO_2_ emissions from coal combustion, and indirect CO_2_ emissions induced by electricity consumption in China’s cement industry. We adopt the mass balance method recommended by the Intergovernmental Panel on Climate Change to account for the emissions (IPCC, 2006).

### 2.2 Emission calculation

#### 2.2.1 Process‐related CO_2_ emissions

The process‐related CO_2_ emissions during cement production can be estimated as the cement or clinker production timed by the related emission factors. As discussed above, most of the previous studies use the cement production to calculate the cement process‐related emissions. These cement production‐based emission accounts may overlook the regional diversity in the cement manufacturing process and cement–clinker ratio. Therefore, the present study calculates the process‐related emissions based on clinker production to achieve more accurate emission accounts of the cement industry; see Equation 2 (IPCC, 2006).

2 $$ \kern0.33em C{E}_{\mathrm{process}}=A{D}_{\mathrm{clinker}}\kern0.33em \times E{F}_{\mathrm{clinker}}. $$

In the above equation, $A{D}_{\mathrm{clinker}}$ refers to the clinker production, while $E{F}_{\mathrm{clinker}}$ is the emission factor for clinker production, that is, the CO_2_ emitted during per unit production of clinker.

The emission factor for cement is collected from Liu et al. (2015), which is 0.4964 tonne CO_2_ per tonne of clinker production.

#### 2.2.2 Direct CO_2_ emissions from coal combustion (coal‐related CO_2_ emissions)

The direct coal‐related CO_2_ emissions are estimated using Equation 3 (IPCC, 2006).

3 $$ \kern0.33em C{E}_{\mathrm{coal}}=A{D}_{\mathrm{coal}}\kern0.33em \times \kern0.33em E{F}_{\mathrm{coal}}=A{D}_{\mathrm{coal}}\kern0.33em \times NCV\times CC\times O. $$

In the above equation, $C{E}_{\mathrm{coal}}$ is the direct coal‐related CO_2_ emissions in the cement production, $A{D}_{\mathrm{coal}}$ (activity data) refers to the coal consumption, and $E{F}_{\mathrm{coal}}$ is the emission factor of coal, which is made up of three components: $NCV$ (net caloric value), $CC$ (carbon content), and *O* (oxygenation efficiency). The parameters are collected from our previous study on China’s coal quality based on an extensive investigation of 4,243 coal mines (Liu et al., 2015). The $NCV$ of coal is 20.60 PJ/mt, the $CC$ is 26.32 tC/TJ (Shan, Guan, Zheng, et al., 2018), and the *O* is 92%. The overall emission factor of coal ($E{F}_{\mathrm{coal}}$) is 0.499 tonne CO_2_ emissions per tonne of coal consumption.

#### 2.2.3 Indirect CO_2_ emissions from electricity consumption (electricity‐related CO_2_ emissions)

The indirect CO_2_ emissions induced by purchased electricity consumption are calculated using Equation 2 (IPCC, 2006).

4 $$ \kern0.33em C{E}_{\mathrm{ele}}=A{D}_{\mathrm{ele}}\kern0.33em \times E{F}_{\mathrm{ele}}. $$

In the equation, $C{E}_{\mathrm{ele}}$ is the electricity‐related CO_2_ emissions in the cement production and $A{D}_{\mathrm{ele}}$ (activity data) refers to the electricity consumption, while $E{F}_{\mathrm{ele}}$ refers to the emission factor. This study uses the regional average electricity emission factors (NDRC, 2009) for each province (see Table Table 1).

**Table 1:** Emission factors of electricity (NDRC, 2009)

Regional grid	Provinces	$E{F}_{\mathrm{ele}}\left(\ \mathrm{kg}/\mathrm{kwh}\ \right)$
North	Beijing, Tianjin, Hebei, Shanxi, Shandong, Inner Mongolia West	1.246
Northeast	Liaoning, Jilin, Heilongjiang, Inner Mongolia East	1.096
East	Shanghai, Jiangsu, Zhejiang, Anhui, Fujian	0.928
Central China	Henan, Hubei, Hunan, Jiangxi, Sichuan, Chongqing	0.801
Northwest	Shaanxi, Gansu, Qinghai, Ningxia, Xinjiang	0.977
South	Guangdong, Guangxi, Yunnan, Guizhou	0.714
Hainan	Hainan	0.917

#### 2.2.4 CO_2_ emissions induced by domestic construction and exports

Cement is mainly used in the construction industry to build new buildings. Therefore, the cement‐related CO_2_ emissions are closely associated with the new investment in the construction industry. In order to better analyze the CO_2_ emissions, we split the total cement‐related emissions into seven parts according to the cement demands of different domestic new building types and export.

We first exclude the export‐related emissions from the total amount. The export‐related emissions are calculated based on the cement clinker export amount, which is collected from the CCA (2009–2014). The remaining CO_2_ emissions are associated with domestic new building’s construction, which can be categorized into six types: residential, manufacturing, infrastructural, commercial, science–education–culture–health, and other buildings. We collect each new building types’ construction outputs from CCA (2009–2014) and calculate the proportion of each type, respectively. We then divide the domestic emissions into six parts according to the proportion of each new building type.

### 2.3 Index decomposition analysis

Understanding the drivers leading to the cement‐related CO_2_ emission peak has practical importance for further emission reduction policymaking. Techniques available for conducting such analyses include structural decomposition analysis (SDA; Rose & Casler, 1996) and IDA (Ang, 2004; Liu, Geng, Lindner, Zhao, et al., 2012), both of which have been extensively applied to quantify the socioeconomic driving factors of dependent variations, such as energy consumption or CO_2_ emissions (Baležentis, Baležentis, & Streimikiene, 2011; Dhakal, 2009; Guan et al., 2018; Mi et al., 2017). SDAs enable us to capture both direct and indirect effects along the entire supply chain on the basis of input–output tables (Meng et al., 2018). This study focuses on the emissions from 1996 to 2016 and uses IDA because of two reasons. First, all the cement‐related CO_2_ emissions are from the sector of nonmetallic products, only a small part of sectors in China’s economy is involved in the supply chain. Second, the time series input–output tables in China are not available. Thus, we use IDA to decompose the cement‐related CO_2_ emissions.

A variety of methods for IDA have been developed, most of which are versions of the Laspeyres index or Divisia index methods. Ang (2004) proposed an LMDI method based on the Divisia index. The LMDI method is the most preferred method, as it passes a number of basic tests for a good index number. The decomposition is perfect, which means that there is no residual term that other methods might produce. The LMDI method can also deal with zero values better than other methods (Ang & Liu, 2007a, 2007b). The LMDI method has the advantages of “*path independence, consistency in aggregation and easily interpreted results*” (Liu, Geng, Lindner, & Guan, 2012; Meng et al., 2016). By using the LMDI method, previous studies have explored the drivers of regional emission growth (Chong, Guan, & Guthrie, 2012; Guan et al., 2018; Liu, 2016a; Liu, Fan, Wu, & Wei, 2007; Wang et al., 2014; Zhang, Li, Zhou, Zhang, & Gao, 2016). This study, therefore, employs LMID to quantify the drivers of CO_2_ emissions’ changes in China’s cement industry. Detailed methods are presented in the Supporting Information.

Four driving factors are defined in Equation 5 to explain the total changes in the cement‐related CO_2_ emissions: the construction industry’s structure, emission intensity, efficiency, and fixed capital formation. The changes in each factor help quantify the change in CO_2_ emissions from the cement’s different usages, environmental effects, technological advancement, and economic growth aspects.

5 $$ \mathrm{CE}\kern0.58em =\sum \limits_iC{E}_i\kern0.33em =\sum \limits_i\frac{C{E}_i}{CE}\kern0.33em \times \frac{CE}{P}\times \frac{P}{F}\times F\kern0.33em =\kern0.33em \ \mathrm{SIEF} $$

In the above equation, $CE$ is the total cement‐related CO_2_ emissions. $C{E}_i$ are emissions induced by different new building types, which reflects the construction industry’s structure in China. We merge the seven types defined in Section 2.2.4 to three for a clear and concise decomposition analysis: residential and commercial buildings; infrastructural buildings (manufacturing buildings, science–education–culture–health buildings); exports; and others. The building types merged together have similar characters. *P* is the cement production, and *F* represents the fixed capital formation in respective years.

The four different factors are:

1. ${\mathrm{S}}_i=C{E}_i/ CE\kern0.33em$ (proportion of CO_2_ emissions, in percentage) measures the share of CO_2_ emitted from cement usage *i*, representing the construction industry’s structural effect;
2. $I\kern0.33em =\kern0.33em CE/P$ (emission intensity, in tonne/tonne) measures the CO_2_ emissions per unit of cement production, representing the environmental impacts in the cement production;
3. $E\kern0.33em =\kern0.33em P/F$ (input efficiency, in tonne/Chinese yuan) measures the cement production per unit of fixed capital formation, representing the technological advancements in the cement production;
4. *F* (fixed capital formation, in Chinese yuan) stands for the economic growth.

### 2.4 Data sources

#### 2.4.1 Cement and clinker production

National Bureau of Statistics ([NBS], 2018) provides the national and provincial cement production from 1996 to 2016. The CCA (2009–2014) publishes the cement production of the country from 1996 to 2013, clinker productions for the country from 2002 to 2013, and the cement–clinker for the provinces from 2005 to 2012. We compare the national and provincial aggregated cement production of NBS and CCA. We find that the difference between the two sources is within ±1% (as shown in Table S1 in the Supporting Information). This demonstrates that the quality of China’s cement statistics is relatively high. In order to achieve more accurate CO_2_ emission accounts for China’s cement industry, we integrate both NBS and CCA’s data and obtain the cement and clinker production data.

At the national level:

1. The cement production from 1996 to 2013 is collected from CCA, and the data from 2014 to 2016 are collected from NBS. The clinker production from 2002 to 2013 is collected from CCA.
2. We then calculate China’s clinker‐to‐cement ratios from 2002 to 2013. The clinker‐to‐cement ratio is calculated as the clinker production divided by the cement production.
3. China’s clinker‐to‐cement ratios from 2014 to 2016 are estimated based on a linear regression of historical ratios and the years (2002–2013); the ratios from 1996 to 2001 are assumed to be the same as those in 2002.
4. The clinker production from 1996 to 2001 and 2014 to 2016 are estimated as the product of clinker‐to‐cement ratios and corresponding cement production volume.

At the provincial level:

1. The provincial cement and clinker production from 2005 to 2012 are collected from CCA, and their cement production from 1996 to 2004 and 2013 to 2016 are collected from NBS.
2. The clinker‐to‐cement ratios of every province from 2005 to 2012 are calculated based on the provinces’ cement and clinker production volume.
3. The clinker‐to‐cement ratios of every province from 2002 to 2004 and 2013 are estimated with the country’s overall ratios and the provinces’ ratios. We assume the provinces have the same change rates in clinker‐to‐cement ratio as those at national level. For example, the 2004 provincial ratios are estimated as $\mathrm{Province}\_\mathrm{Rati}\ {\mathrm{o}}_{2004}=\frac{\ \mathrm{National}\_\mathrm{Rati}\ {\mathrm{o}}_{2004}}{\ \mathrm{National}\_\mathrm{Rati}\ {\mathrm{o}}_{2005}}\kern0.33em \times \mathrm{Province}\_\mathrm{Rati}\ {\mathrm{o}}_{2005}$. Similarly, the 2013 provincial ratios are estimated based on 2012 provincial ratios and 2012–2013 national ratios.
4. The clinker‐to‐cement ratios of every province from 1996 to 2001 and 2014 to 2016 are considered the same as those in 2002 and 2013, assuming the ratios remain unchanged.
5. The clinker production of every province from 1996 to 2004 and 2013 to 2016 are estimated as the product of clinker‐to‐cement ratios and corresponding cement production volume.

The cement and clinker productions, and the clinker‐to‐cement ratios of China (for both the nation and provinces) are presented in the Supporting Information (Tables S2–S4, respectively).

#### 2.4.2 Coal and electricity consumptions

The CCA (2009–2014) published the total energy consumption and coal consumption of each province from 2005 to 2012. We estimate the provinces’ energy consumption for the remaining years assuming that the energy intensities (per unit of cement production energy consumption) remain the same. The electricity consumption is estimated as the total energy consumption minus the coal consumption, as coal and electricity are the primary energies used in China’s cement industry (CCA, 2009–2014).

The coal and electricity consumption in China and each province’s cement industry are presented in the Supporting Information (Tables S5 and S6, respectively).

#### 2.4.3 Other indexes used in IDA

The fixed capital formations of China are collected from the official database of NBS (2018) and are consistent with the latest published statistical yearbooks in China. We convert all the economic data to the 2002 constant price to eliminate the effects of price fluctuation. The cement and clinker exports are collected from CCA (2009–2014). The construction output of each new building type are also collected from CCA (2009–2014). The indexes are listed in Table S7 in the Supporting Information.

## RESULTS AND DISCUSSION

### 3.1 Emissions from China’s cement industry

**Figure 2:**
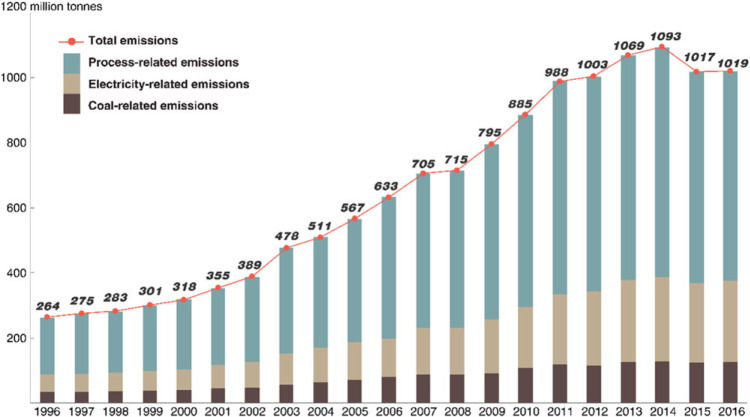
CO_2_ emissions from China’s cement industry

Figure 2 presents the overall CO_2_ emissions from China’s cement industry. The total cement‐related CO_2_ emissions in 2016 reached 1,019 Mt, accounting for 11.06% of China’s total CO_2_ emissions (9,217 Mt in 2016; Shan, Guan, Zheng, et al., 2018). The total cement‐related CO_2_ emissions in China peaked in 2014, at 1,093 Mt.

Before their peak in 2014, the cement‐related emissions can be divided into three phases based on different growth characteristics. (a) Stable growth (1996–2002): the cement‐related CO_2_ emissions increased stably from 264 to 389 Mt during the period, with an average growth ratio of 6.6% per year. (b) Rapid growth (2002–2011): stimulated by joining the WTO in 2002, China’s cement production rapidly increased beginning in 2002. The cement‐related CO_2_ emissions surged as well. The average emission growth ratio reached 10.9% per year. The emission growth temporarily stagnated in 2008 due to the global financial crisis and the depressed cement market demand. (c) Slow growth (2011–2014): beginning in 2012, the growth of cement‐related emissions slowed (3.4% per year averagely) due to economic cooling.

The growth of cement‐related CO_2_ emissions was coupled with the trends of China’s GDP growth (NBS, 2018). This correlation can be explained by the relationships among the emissions, production/demand, and economic inputs in the cement industry: (a) the CO_2_ emissions are calculated based on the clinker/cement production; (b) the cement is a building material that is not easy to preserve, and the plants normally produce cement based on the market demand or purchasing orders; and (c) cement demand is closely associated with the number of new buildings, which is primarily stimulated by the country’s fixed capital formation. In this way, it could be inferred that the cement‐related CO_2_ emissions were highly driven by the country’s economic gains.

After the three increasing phases, China’s cement‐related emissions peaked in 2014. In 2015, the overall cement‐related emissions appeared to decline for the first time. The emissions decreased by 76 Mt (or 7.5%) in 1 year. This decline was mainly caused by a decrease in cement production. The total cement production decreased from 2,492 Mt in 2014 to 2,359 in 2015.

By investigating the detailed emission mix, we find that the process‐related CO_2_ emissions were the primary source of cement‐related emissions in China, accounting for 63.1% in 2016. The coal‐ and electricity‐related emissions accounted for the remaining 12.3% and 21.6%, respectively. The shares of emissions caused by different sources have changed slightly over the past 20 years. The process‐related emissions decreased from 67.0% in 1996 to 63.1% in 2016 (with an average value of 66.1%), while the emissions induced by purchased electricity consumption increased from 20.2% in 1996 to 24.4% in 2016 (with an average value of 21.6%). The coal‐related emissions remained stable at approximately 12.3% over the 20‐year study period. As we use the same emission factor for emission accounts over years, the unchanged emission mix implies that the energy intensity (energy consumption per units of cement production) has not changed greatly, that is, the energy efficiency and utilization technology in China’s cement production remained at the same level during the past 20 years.

**Figure 3:**
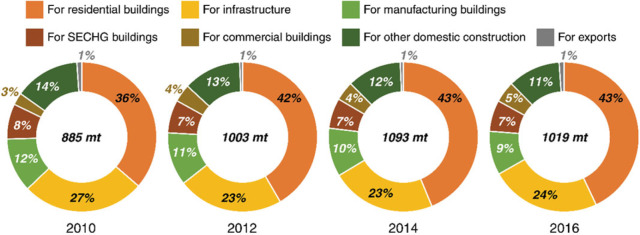
Cement‐related CO_2_ emissions by new building types *Note*: SECHG is short for science–education–culture–health–government

Figure 3 presents the CO_2_ emissions induced by the cement consumption in each new building type in selected study years. The results show that the emissions induced by cement produced for different new building types remain stable over time, implying a relatively stable structure in China’s construction industry. Residential buildings are the major contributors to the cement‐related emissions, accounting for approximately 40%, followed by infrastructure (approximately 25%) and the manufacturing plants (approximately 10%). Science–education–culture–health–government (SECHG) and commercial buildings emitted approximately 7% and 4% of total emissions, respectively. The emissions induced by the cement export accounted for only 1% of the total emissions due to the small export volume. Cement is expensive for long‐distance transport due to its short shelf life and high density. It is much more cost‐effective to self‐produce cement. China exports less than 10 Mt of cement and clinker to Africa and the USA/EU every year, which is a very small proportion of the 2,410 Mt cement and 1,295 Mt clinker produced.

This study also calculates the emission intensity of cement production. The emission intensity is defined as CO_2_ emissions per unit of cement production. We find that the emission intensity of the cement production slightly decreased during the past two decades from 0.54 (in 1996) to 0.42 tonne CO_2_ per tonne of cement production (in 2016).

The process‐related, direct coal‐related, and indirect electricity‐related CO_2_ emissions of cement industries at both the national and individual province levels are presented in the Supporting Information (Tables S8–S10, respectively). The total cement‐related CO_2_ emissions are summarized in Table S11 in the Supporting Information.

### 3.2 Regional differences in cement production

**Figure 4:**
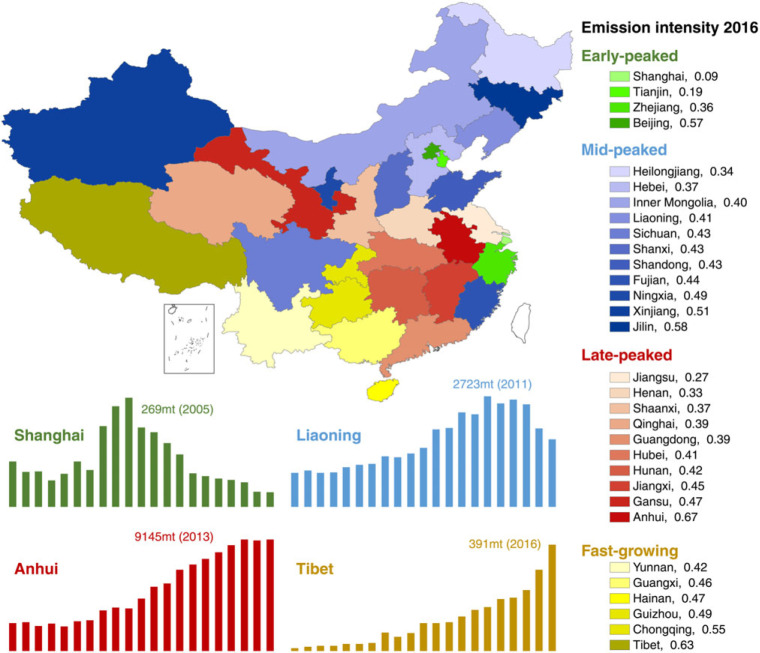
Provincial CO_2_ emissions from the cement industry *Note*: The number following the province name presents the emission intensity of the province in 2016. The early‐peaked, mid‐peaked, late‐peaked, and fast‐growing provinces are shown in green, blue, red, and yellow, respectively

Despite the overall cement‐related emissions having peaked in China, considering the regional differences in China, not all provinces have peaked their cement‐related emissions yet. Certain provinces are still in the rapidly increasing stage. We classify the 31 provinces (excluding Taiwan, Hong Kong, and Macao due to a lack of data) into four groups at different peak stages, as shown in Figure 4.

The early‐peaked group includes the four most developed provinces in China: Beijing, Shanghai, Tianjin, and Zhejiang. These four provinces peaked their cement‐related emissions around 2006. The early‐peaked provinces have the lowest emission intensity (0.35 tonne CO_2_ per tonne cement production) among the four province groups. The mid‐peaked group includes 11 provinces, most of them located in northern and northeast China. These provinces peaked their cement‐related emissions around 2012. The average emission intensity of these 11 mid‐peaked provinces is 0.43 tonne CO_2_ per tonne cement production. Another 10 provinces belong to the late‐peaked group, which peaked their cement‐related emissions around 2014. The fast‐growing provinces are still continuously increasing their cement‐related emissions. Yunnan, Guangxi, Hainan, Guizhou, Chongqing, and Tibet belong to this group. The fast‐growing group, therefore, has the highest average emission intensity of 0.47 tonne CO_2_ per tonne cement production.

### 3.3 Production capacity shifts among provinces

The provinces’ different technical levels could affect their emission peak stages and emission intensities. The early‐peaked provinces have advanced technologies, which consume less energy during the cement production. However, the discrepancies can be largely explained by the clinker trade among provinces, which can be shown from the clinker‐to‐cement ratios of the provinces.

**Figure 5:**
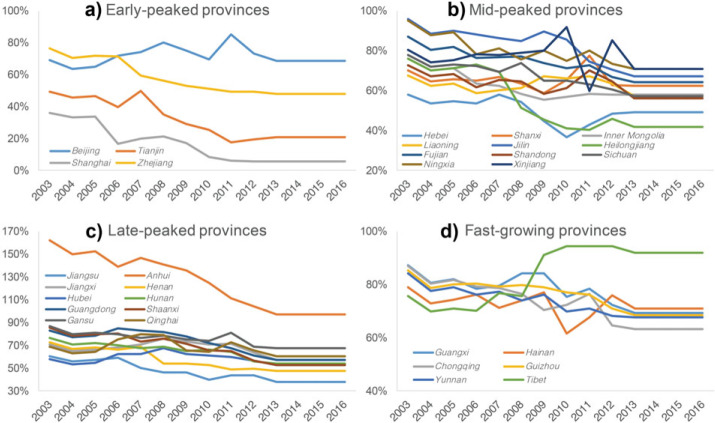
Clinker‐to‐cement ratio changes in provinces

We present the provinces’ clinker‐to‐cement ratios in Figure 5. The overall national ratio decreased uniformly from 72.4% in 2002 to 53.7% in 2016 with an average value of 65.0%. A higher ratio indicates a larger clinker production and a smaller cement production, whereas a smaller ratio indicates a smaller clinker production but a larger cement production.

Figure 5a shows that the early‐peaked provinces’ clinker‐to‐cement ratios decreased markedly since 2006, especially Shanghai, Tianjin, and Zhejiang. The decline of the clinker‐to‐cement ratios implies that these provinces reduced their clinker production and imported clinker from other provinces to produce cement. As most of the CO_2_ is emitted during the clinker production process (Worrell et al., 2001), a clinker‐outsourcing policy effectively reduced the provinces’ overall CO_2_ emissions. Taking Shanghai as an example, its clinker‐to‐cement ratio decreased considerably from 33.9% (in 2005) to 16.6% (in 2006) and further to 8.5% (in 2010). Shanghai massively reduced its clinker production two times, in 2006 and 2010. Its clinker production reduced by 1.7 Mt (or 46.9%) and 0.8 Mt (or 57.5%), respectively, in the 2 years. However, the cement production of Shanghai remained stable at the same time (10.5 Mt in 2005, 11.3 Mt in 2006, and 6.7 Mt in 2010), which illustrates that Shanghai reduced its clinker production and imported the clinker from other regions. As a result, the emission intensity of Shanghai is the lowest among all the provinces, with 0.09 tonne CO_2_ per tonne cement production.

Similarly, Figure 5b shows that most of the mid‐peaked provinces’ clinker‐to‐cement ratios decreased from 2007 to 2010, such as Heilongjiang and Jilin. As for the late‐peaked provinces (shown in Figure 5c), there is no remarkable sudden drop in the clinker‐to‐cement ratio. Despite the ratio of Anhui decreasing continuously since 2003, this decrease is mainly caused by the increase in Anhui’s cement production rather than the decrease in its clinker production. Anhui’s clinker production kept increasing until 2014, which caused Anhui’s cement‐related emissions to peak at 9,145 Mt in 2014. Anhui is the largest clinker production base in China. In 2016, the clinker production of Anhui was 121 Mt, accounting for 9.3% of the national production. However, Anhui’s cement production accounted for only 5.6% (or 136 Mt) of the national total production, illustrating that Anhui exports large amounts of clinker to other regions. The average clinker‐to‐cement ratio of Anhui was as high as 134.5% over the past 20 years, which is more than twice the national average level of 61.7%.

Figure 5d presents the clinker‐to‐cement ratios of the six fast‐growing provinces. We find that most of the provinces had a relatively stable ratio during the past 20 years. It is worth noting that the clinker‐to‐cement ratio of Tibet increased suddenly from 75.7% (in 2008) to 91.0% (in 2009), mainly due to the increase of Tibet’s clinker production. Tibet enlarged its cement production capacity since 2000 and formed the integrated company “Huaxin Cement (Tibet)” in 2009 (Liu, 2009; Shan et al., 2017).

To summarize, certain developed provinces have closed or outsourced their clinker production to other less developed regions in the past 10 years. Such outsourcing may be effective in reducing the developed provinces’ emissions in the short term, but it may not be beneficial to the country’s overall emissions reduction (Shan, Guan, Hubacek, et al., 2009). The developed provinces usually have more advanced technologies than those in less developed regions, that is, low emission intensities. Moving the production capacities from regions with advanced technologies to less developed regions will, therefore, increase the country’s overall emissions. Thus, greater attention should be paid to these key cement production provinces, such as Tibet, which will continue developing its cement production in the future (Tibet autonomous region government, 2009), and Anhui, which is the largest cement production base in China.

### 3.4 Changes in the drivers of the cement‐related CO_2_ emissions in China

**Figure 6:**
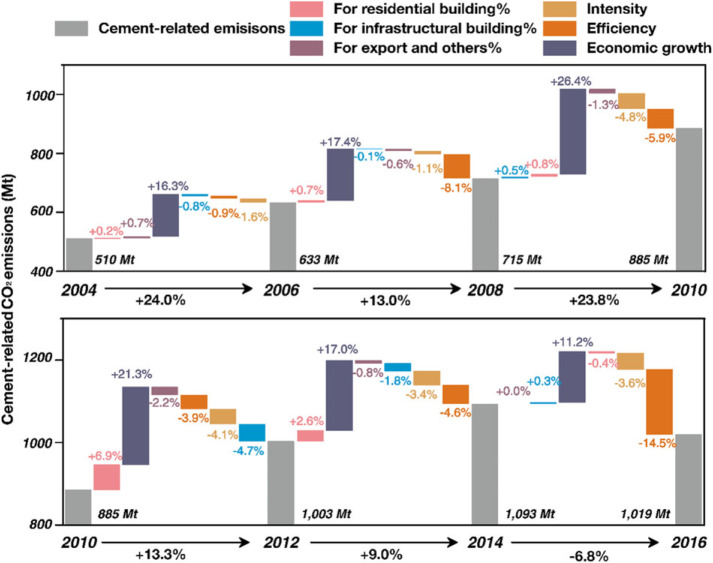
Driving forces of the changes in cement‐related CO_2_ emissions *Note*: The residential buildings in the figure include both residential and commercial buildings; the infrastructural buildings in the figure include infrastructural, manufacturing, and SECHG buildings

Figure 2 shows that the total cement‐related CO_2_ emissions started to grow rapidly since 2002 after China joining the WTO and then peaked in 2014. Understanding of the changes in the drivers of the cement‐related CO_2_ emissions since 2002, especially before and after the peak point (2014), has great policy implications for emissions control in the cement industry. By employing the LMDI method, this study decomposes the changes in China’ cement‐related CO_2_ emissions from 2002 to 2016 into four factors: the construction industry’s structure, emission intensity, efficiency, and economic growth. The first factor, “construction industry’s structure,” is measured by three indicators: the emission proportion induced by cement produced for new residential buildings, for new infrastructural buildings, and for a new proportion of export and others. The results are shown in Table S7 in the Supporting Information and Figure 6.

The results show that economic growth (fixed capital formation) was the major source of the cement‐related emissions growth for a long time, especially during the period 2008–2010. The factor contributed 26.4% of the emissions growth during the period if other factors had remained constant. This growth was mainly stimulated by the “Economic Stimulus Plans” in China. The Chinese government invested four trillion Chinese yuan (approximately 586 billion U.S. dollars) in response to the global financial crisis in 2007. The money was used to expand domestic demand. Local governments in China built several manufacturing plants and infrastructure at the time, such as power plants, cement plants, undergrounds, and high‐speed railways. After 2010, the contribution of economic growth decreased slightly to 21.3% (2010–2012), 17.0% (2012–2014), and 11.2% (2014–2016) due to the gradually weakened stimulation of the “Economic Stimulus Plans,” but economic growth was still the primary power of the cement‐related emission increase.

Emission intensity and efficiency were two major factors offsetting the growth of cement‐related CO_2_ emissions. Efficiency offset 14.5% of the emission growth from 2014 to 2016. The efficiency decreased by 14.3% (from 1.19 to 1.02 tonnes cement production per 10,000 yuan fixed capital formation). This change implies a technical improvement in cement production, as the plants produced more cement with less economic inputs. As for the intensity factor, the emission intensity of cement production decreased slightly during the past two decades from 0.54 (in 1996) to 0.42 tonne CO_2_ per tonne cement production (in 2016), as discussed above. As a result, the emission intensity consistently offset 3.5–4.5% of the emissions’ growth since 2008.

The changes in the construction industry’s structure had significant influences on the related emissions changes since 2010. The proportion of newly constructed residential buildings first increased the cement‐related emissions by 6.9% and 2.6% during the periods 2010–2012 and 2012–2014; then, it offset the emission growth by 0.4% during 2014–2016. This pattern may be affected by the changes in housing prices. According to the China Real Estate Index System (CREIS), China’s housing prices increased by 19.0% from 2010 to 2014, then decreased by 1.1% in 2015 (China Index Academy, 2009). The CREIS housing prices were calculated as the median housing price of 100+ Chinese cities. Considering the delayed action in the construction industry, the construction plan is normally made based on the previous year’s housing price. Therefore, the negative contribution of the proportion of newly invested residential buildings in cement‐related emissions may be influenced by the housing price decreasing in 2015.

In contrast, the proportion of newly invested infrastructural buildings has acted as a negative contributor to the cement‐related emissions since 2010. This factor offset the emissions by 4.7% and 1.8% from 2010 to 2012 and 2012 to 2014, respectively. Stimulated by the “Economic Stimulus Plans,” China has faced the problem of overcapacity since 2010 (Yang & Yu, 2011; Yongding, 2009). The country then reduced its investments in infrastructure construction.

Cement exports had little influence on the emission changes, except for the period 2010–2012. The decrease in cement exports in 2012 was mainly caused by the shrinking of the USA/EU markets, which were affected by the global final crisis (Hefei Cement Research & Design Institute, 2009).

Comparing the contributions of each factor between 2012–2014 and 2014–2016, this study finds that the improvement in efficiency and the reduction in residential building construction were the two major reasons causing the cement‐related CO_2_ emissions’ decrease since 2014.

## CONCLUSIONS AND POLICY IMPLICATIONS

The cement industry is the primary source of process‐related CO_2_ emissions in China and worldwide. This industry contributed 11% of the total emissions in China. As the cement industry is regarded as one of the key energy‐intensive manufacturing sectors, greater attention should be paid to its sustainable production and emission control. Understanding the emission characteristics of China’s cement industry and the drivers of the emission changes is an essential foundation for related policymaking.

Our study first calculates the CO_2_ emissions from the cement industries in China. The emissions include process‐related emissions, direct emissions from fossil fuel combustion, and indirect emissions from purchased electricity. This study then calculates the cement‐related emissions of 31 provinces in China and cluster the provinces into four groups based on different peak stages. Finally, the study calculates the drivers of the changes in cement‐related CO_2_ emissions with the LMDI decomposition analysis.

The emission accounts of China’s cement industry finds that: (a) the total cement‐related CO_2_ emissions in China peaked in 2014 at 1,093 Mt; (b) the emissions growth was coupled with the trend in China’s GDP growth, which implied that the emissions were highly driven by the country’s economic gains; (c) the process‐related emissions were the primary source of the cement‐related emissions in China, accounting for 63.1% in 2016, and the emission mix of the process‐related, direct, and indirect emissions has changed slightly over the past 20 years; and (d) the emissions induced by different new building types also remained stable over the time, implying a relatively stable structure in China’s construction industry. Residential buildings are the major contributor to the overall emissions, followed by infrastructure and manufacturing plants.

Then, our regional analysis of China’s cement‐related CO_2_ emissions has significant implications to manage the cement industry and production capacities of every province; and optimize the whole country’s cement production and utilization. Four developed provinces belong to the early‐peaked group with the lowest emission intensities, whose cement‐related emissions peaked around 2006, and 11 provinces are classified as the mid‐peaked group, which peaked their emissions around 2012. Another 10 provinces that peaked their emissions around 2014 are grouped as the late‐peaked provinces. The remaining six provinces are still increasing their cement‐related emissions due to their increasing production capacities. The analysis of provincial clinker‐to‐cement ratio finds that the above discrepancies in provincial peak stages can be explained by the capacity outsourcing among provinces. The developed provinces have closed or outsourced their clinker production capacities to other less developed regions. Despite the similar outsourcing can be found in other industries (Shan, Guan, Hubacek, et al., 2018), this is certainly not a sustainable development pathway. Such outsourcing may effectively reduce the developed provinces’ emissions in the short term, but it may not be beneficial to the country’s overall emissions reduction. Moving the production capacities from regions with advanced technologies to less developed regions will increase the country’s overall emissions. Stimulated by the “Economic Stimulus Plans” and other development strategies, China is still in the stage of expanding infrastructure construction, especially in the currently backward cities. The central and local governments should coordinate the management of cement production in various provinces and cities, avoid overcapacity in the region, and large‐scale unnecessary cement production outsourcing and shift among provinces in China. Also, backward areas should learn from developed regions to improve their cement production efficiency and reduce unit output emissions.

The decomposition analysis of the cement‐related CO_2_ emissions illustrates the driving forces hidden behind the emission changes. Economic growth was the major source of the growth in cement‐related emissions for a long time, especially in the period 2008–2010. The emissions intensity and efficiency were two major factors offsetting the growth of cement‐related CO_2_ emissions. Since 2014, the efficiency played a more importation role in reducing the emissions, which lead to the decoupling of economic growth and cement‐related CO_2_ emissions (Wu et al., 2018). As more and more studies have found that China is entering a phase of decoupling economic growth from carbon emissions, efficiency gains can further reduce the emissions in the future. For example, the cement plants may consider using a cleaner energy mix to reduce the emission intensity or use low‐cost energies (nonrecycled plastics and paper as alternative fuels) to improve the efficiency (Bourtsalas, Zhang, Castaldi, & Themelis, 2018; Huh, Lee, Shin, Lee, & Jang, 2017). These low‐cost energies should be used in a clean way. Cleaner production techniques should also be developed and applied to the cement plants in China, such as the “calcium looping CO_2_ capture” (Schakel et al., 2018).

Apart from the economic driver, the changes in the structure of the construction industry also had significant influences on the related emissions changes since 2010. The proportion of new residential buildings became a negative contributor to emissions growth since 2014, mainly influenced by the housing price fluctuation in China. However, the change in house prices is highly sensitive. The governments should plan the construction industry and new building construction to eliminate the erratic effects of housing prices on cement production. In this way, the overcapacity and waste of cement production can be avoided.

In the future, we will conduct a more detailed investigation of each province’ cement industry to analyze the impact of the cement‐related CO_2_ emissions. Also, further study could use life cycle assessment (LCA) and carbon footprint analysis to provide a more detailed evaluation of the emission performance of cement production and consumption. The LCA and carbon footprint analysis can allocate the cement‐related environmental performance to the end use in the construction industry (Fořt & Černý, 2018). Then, the emissions from the cement industry can be controlled from both the production and demand perspectives.

## Supplementary Information

**Supporting Information S1**: This supporting information includes details on index decomposition analysis (IDA)–LMDI with figures and tables.

## Footnotes

1 All the data and results can be downloaded freely from the “China Emission Accounts and Datasets (CEADs)” at http://www.ceads.net and have been deposited in the data repository, Figshare. (Shan, Zhou, Meng, et al. 2009) for re‐use.
