# Supplementary material for: Peak cement‐related CO2 emissions and the changes in drivers in China
Source: J Ind Ecol. 2019 Feb 8;23(4):959–71. doi: 10.1111/jiec.12839 (PMC13065526; doi:10.1111/jiec.12839)
Supplement: Supplementary file 1 — Supporting Information S1: This supporting information includes details on index decomposition analysis (IDA)–LMDI with figures and tables. [file 44498_2019_2304017_MOESM1_ESM.pdf]

SUPPORTING INFORMATION FOR:

Shan, Y., Y. Zhou, J. Meng, Z. Mi, J. Liu, and D. Guan. 2019. Peak cement-related CO2 emissions and the changes in drivers in China. *Journal of Industrial Ecology.*

Summary

This supporting information includes details on Index Decomposition Analysis (IDA) – LMDI with figures and tables.

1 Index Decomposition Analysis (IDA) – LMDI

The calculations of cement-related CO_2 emission contributions are based on IDA. According to Ang (2005), let V be an environmentally related aggregate. Assume that there are n factors driving the changes in V over time and that each is associated with a quantifiable variable, whereby there are n variables: X_1, X_2, …, X_n. Let subscript i be a sub-category of the aggregate, and V_i is expressed as the product of $X_{1,i}$, $X_{2,i}$, …, $X_{n,i}$. The general IDA identity is given in Equation 1.

$$\mathrm{V} = \sum_i V_i = \sum_i X_{1,i} \cdot X_{2i} \cdot \ldots \cdot X_{n,i} \qquad \textit{Equation 1}$$

In the additive approach, the difference in the aggregate between period 0 and T is decomposed, as shown in Equation 2.

$$\Delta \mathrm{V}_{total} = V^T - V^0 = \Delta V_{x_1} + \Delta V_{x_2} + \cdots + \Delta X_{x_n} \qquad \textit{Equation 2}$$

The sum of the absolute change driven by each variable should be equal to the total absolute change of the aggregate. The terms on the right-hand side of Equation 2 are the effects of each factor in Equation 1 if the other factors remain constant.

Changes in CO_2 emissions from cement production can be studied by quantifying the impacts of changes in four factors: the construction industry's structure, emission intensity, efficiency, and fixed capital formation. The changes in each factor help quantify the change in CO_2 emissions from the cement's different usages, environmental effects, technological advancement, and economic growth aspects. The IDA identity in Equation 1 can be written as follows:

$$\mathrm{CE} = \sum_i CE_i = \sum_i \frac{CE_i}{CE} \times \frac{CE}{P} \times \frac{P}{F} \times F = \mathrm{SIEF} \qquad \textit{Equation 3}$$

In the above equation, CE is the total cement-related CO_2 emissions. The total emissions are divided into three parts (CE_i) according to the cement consumption in different new-building types, which reflects the construction industry's structure in China: for residential and commercial buildings; for infrastructural, manufacturing, science-education-culture-health buildings; and for exports and other buildings. We use the construction area and the output of each new-building type to divide the total emissions. P is the cement production, and F represents the fixed capital formation in respective years.

As shown in Equation 3, the total changes in the cement-related CO_2 emissions are represented by quantifying the contributions driven by the four different factors described above:

1) $S_i = CE_i/CE$ (proportion of CO_2 emissions, in %) measures the share of CO_2 emitted from cement usage i, representing the construction industry's structural effect;

2) $\mathrm{I} = \mathrm{CE}/\mathrm{P}$ (emission intensity, in ton/ton) measures the CO_2 emissions per unit of cement production, representing the environmental impacts in the cement production;
3) $\mathrm{E} = \mathrm{P}/\mathrm{F}$ (input efficiency, in ton/Chinese yuan) measures the cement production per unit of fixed capital formation, representing the technological advancements in the cement production;
4) F (fixed capital formation, in Chinese yuan) stands for the economic growth.

The additive form provides direct information about the magnitude of emission changes by decomposed factors, and the formula for the additive approach is as follows:

$$\Delta CE_{tot} = CE^{T} - CE^{0} = \Delta C_{str} + \Delta C_{int} + \Delta C_{eff} + \Delta C_{eco}$$ *Equation 4*

The subscripts str, int, eff, and eco denote the impacts concerned with the construction industry's structural effect, emission intensity, efficiency, and economic growth aspects, respectively. The effect of a factor is computed by letting that factor change over time with all the other factors remaining at their respective base year values (Ang 2004).

According to Ang (2005), the LMDI is the preferred method since it avoids the allocation of unexplained residual terms, which makes the results simple to interpret. This method is also consistent in aggregation, which means that industry activities can be grouped into sub-groups for further effect estimation. Therefore, the LMDI method was applied in the present study. The effect of decomposition factors on the right-hand side of Equation 4 are quantified by the following equations by using the LMDI method:

$$\Delta C_{str} = \textstyle\sum_i w_i * \ln(S^T/S^0)$$

$$\Delta C_{int} = \textstyle\sum_i w_i * \ln(I^T/SI^0)$$

$$\Delta C_{eff} = \textstyle\sum_i w_i * \ln(E^T/E^0)$$

$$\Delta C_{eco} = \textstyle\sum_i w_i * \ln(F^T/F^0)$$

Equation 5

w_i in Equation 5 is calculated in Equation 6.

$$w_i = (CE_i^T - CE_i^0)/(lnCE_i^T - lnCE_i^0)$$ *Equation 6*

2 Figure

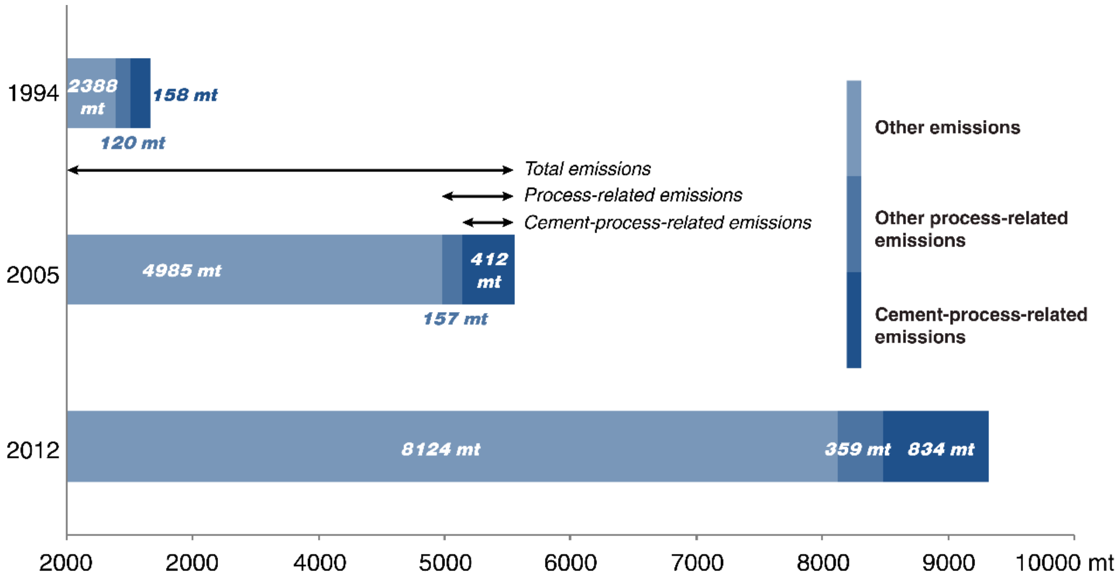

Figure S1 CO_2 emissions from China's national communications on climate change

Note: The National Communication 2012 presented only the total amount of process-related emissions from non-metal production, therefore, the 834 Mt of process-related emissions in 2012 in the figure include the emissions from other non-metal industrial processes.

3 Tables

Table S1 Comparisons of cement production of NBS and CCA (10 thousand tonnes, %)

	National production (NBS)	Provincial aggregated production (NBS)	CCA production	Difference between NBS and CCA production
1996		49899.51	49,212.00	
1997		51173.8	51,276.00	
1998		51392.43	52,706.00	
1999		55307.55	56,105.00	
2000		58319.29	59,319.00	
2001	66,103.99	66103.99	66,104.00	0.00%
2002	72,500.00	72181.26	72,535.00	0.05%
2003	86,208.11	86208.11	86,271.00	0.07%
2004	96,681.99	96681.99	97,300.00	0.64%
2005	106,884.79	106884.79	106,885.00	0.00%
2006	123,676.48	123676.48	123,611.00	-0.05%
2007	136,117.25	136117.26	136,117.00	0.00%
2008	142,355.73	138838.3	142,010.00	-0.24%
2009	164,397.78	164397.77	164,863.00	0.28%
2010	188,191.17	188191.17	187,919.00	-0.14%
2011	209,925.86	209925.86	209,395.00	-0.25%
2012	220,984.08	220984.08	221,339.00	0.16%
2013	241,923.89	241613.61	243,476.00	0.64%
2014	249,207.08	249207.08		
2015	235,918.83	235918.83		
2016	241,030.98	241030.98		

Table S2 Cement production of China and its provinces (10 thousand tonnes)

	**1996**	**1997**	**1998**	**1999**	**2000**	**2001**	**2002**	**2003**	**2004**	**2005**	**2006**
**China**	49212	51276	52706	56105	59319	66104	72535	86271	97300	106885	123611
**Beijing**	657	702	783	815	841	809	888	1000	1222	1184	1229
**Tianjin**	205	209	188	261	272	339	380	451	526	519	609
**Hebei**	3360	3769	3986	4193	4775	4878	5798	6816	7634	7686	8625
**Shanxi**	1290	1423	1431	1448	1214	1573	1688	1950	2401	2311	2681
**Inner Mongolia**	394	467	499	558	641	698	715	949	1291	1632	2211
**Liaoning**	1719	1833	1706	1736	1988	2101	2156	2442	2512	2681	3294
**Jilin**	600	628	644	669	772	907	894	1120	1331	1719	1799
**Heilongjiang**	646	711	763	863	919	966	962	1115	1167	1214	1483
**Shanghai**	438	339	339	255	317	434	353	745	977	1045	1131
**Jiangsu**	3985	4040	3955	4441	4678	5247	6065	7831	8861	9681	10976
**Zhejiang**	3498	3437	3524	3850	4330	4791	5771	7199	8829	9129	9952
**Anhui**	2238	2316	1978	2164	1938	2372	2415	3075	3487	3353	4580
**Fujian**	1484	1525	1496	1852	1540	1762	1707	2402	2398	2792	3399
**Jiangxi**	1048	1050	1162	1334	1488	1608	1976	2526	3114	3701	4300

**Shandong**	5546	5842	5550	6035	6659	7287	8279	9942	12959	14426	16670
**Henan**	3045	3424	3929	3856	3787	4686	4503	4726	5329	6487	7605
**Hubei**	1762	2098	2328	2242	2503	2797	2963	3448	3655	4486	5203
**Hunan**	2269	2213	2398	2307	2437	2762	2760	3137	3407	3742	4589
**Guangdong**	5210	5158	5222	5690	5973	6018	7479	7536	7732	8229	9704
**Guangxi**	1907	1897	2060	2092	2236	2140	2413	2667	2795	3306	3655
**Hainan**	181	188	229	292	320	313	349	398	428	446	585
**Chongqing**	770	979	1204	1215	1427	1699	1759	2039	2005	2226	2619
**Sichuan**	3009	2356	2526	2629	2814	3162	3311	4063	3880	4480	5060
**Guizhou**	515	633	574	721	797	1204	1127	1592	1439	1685	1907
**Yunnan**	1136	1344	1598	1646	1538	1641	1717	2054	2315	2833	3306
**Tibet**	23	32	38	40	50	50	59	125	102	128	167
**Shaanxi**	903	1168	881	1004	1006	1493	1335	1829	2033	2165	2515
**Gansu**	581	604	646	715	736	892	1086	1162	1335	1416	1454
**Qinghai**	75	79	104	118	126	176	265	307	343	371	371
**Ningxia**	163	182	232	252	285	319	376	494	584	568	710
**Xinjiang**	555	629	734	814	910	981	986	1129	1208	1245	1224

	**2007**	**2008**	**2009**	**2010**	**2011**	**2012**	**2013**	**2014**	**2015**	**2016**
**China**	136117	142010	164863	187919	209395	221339	243476	249207	235919	241031
**Beijing**	1169	885	1080	1049	921	884	908	704	554	510
**Tianjin**	615	556	700	832	940	849	959	1071	778	789
**Hebei**	9478	9264	11012	12772	14497	13153	12846	10721	9126	9899
**Shanxi**	2781	2483	2793	3670	4091	5084	5139	4832	3786	3852
**Inner Mongolia**	2871	3262	4478	5470	6483	6072	6487	6405	5831	6298
**Liaoning**	3912	4125	4827	4786	5785	5513	6076	5821	4568	4011
**Jilin**	2083	2342	2764	3080	3792	3248	3417	3706	3325	2765
**Heilongjiang**	1744	2131	2643	3592	4368	3992	4102	3715	3112	3381
**Shanghai**	959	808	783	671	804	800	756	686	434	418
**Jiangsu**	11850	13249	14728	15685	14996	16929	18166	19496	18056	18038
**Zhejiang**	10409	10218	10873	11329	12166	11594	12576	12413	11331	10848
**Anhui**	5402	6172	7138	8069	9548	11022	12286	12982	13208	13584
**Fujian**	4500	4653	5482	5919	6792	7271	7967	7779	7787	8106
**Jiangxi**	5096	5352	6168	6263	6857	7584	9299	9849	9458	9553
**Shandong**	14987	14142	14491	14899	15035	15480	16364	16553	15249	16156
**Henan**	9384	10358	11876	11657	13789	14913	16911	17331	16676	15672
**Hubei**	5639	5899	7037	9004	9480	10392	11135	11424	11145	11601
**Hunan**	5683	6168	7685	8822	9341	10591	11401	12187	11680	12240
**Guangdong**	9896	9768	10160	11611	12682	11504	13533	14812	14530	15081
**Guangxi**	4350	5246	6499	7517	8724	10000	10993	10752	11144	12035
**Hainan**	633	661	939	1264	1518	1675	2004	2152	2225	2228
**Chongqing**	3000	3271	3641	4621	5004	5571	6198	6703	6840	6790
**Sichuan**	6376	6280	9100	12882	14486	13487	14055	14661	14091	14616

**Guizhou**	2059	2076	2675	3810	5296	6760	8252	9598	9941	10799
**Yunnan**	3569	4110	5022	5786	6772	8027	9192	9663	9436	11104
**Tibet**	160	169	188	219	232	287	298	342	468	623
**Shaanxi**	3175	3672	4532	5513	6575	7648	8670	9159	8579	7264
**Gansu**	1540	1635	1825	2425	2753	3658	4461	4935	4764	4640
**Qinghai**	437	462	611	811	1045	1412	1852	1870	1768	1895
**Ningxia**	846	903	1072	1422	1460	1618	1942	1804	1750	1985
**Xinjiang**	1514	1691	2040	2471	3164	4323	5230	5082	4278	4250

Table S3 Clinker production of China and its provinces (10 thousand tonnes)

	1996	1997	1998	1999	2000	2001	2002	2003	2004	2005	2006
**China**	35629	37124	38159	40620	42947	47859	52515	65703	68437	76472	87328
**Beijing**	421	451	506	525	548	524	578	685	776	767	879
**Tianjin**	94	96	87	121	127	158	177	222	239	241	242
**Hebei**	1815	2039	2174	2279	2620	2665	3181	3936	4084	4196	4623
**Shanxi**	839	927	940	948	802	1035	1116	1357	1548	1520	1742
**Inner Mongolia**	278	330	356	396	459	498	512	716	902	1164	1406
**Liaoning**	1080	1153	1082	1097	1269	1335	1376	1639	1563	1702	1930
**Jilin**	535	561	579	600	699	818	809	1067	1176	1549	1584
**Heilongjiang**	456	503	544	613	659	689	690	841	816	866	1084
**Shanghai**	147	114	115	86	108	147	120	267	324	354	188
**Jiangsu**	2247	2281	2251	2520	2679	2992	3474	4720	4949	5517	6476
**Zhejiang**	2486	2445	2527	2753	3125	3443	4165	5468	6214	6556	7087
**Anhui**	3370	3490	3005	3278	2963	3611	3693	4948	5198	5101	6359
**Fujian**	1201	1236	1222	1508	1266	1443	1403	2078	1922	2284	2592
**Jiangxi**	688	690	770	881	992	1068	1318	1773	2025	2456	2918
**Shandong**	3741	3945	3778	4095	4561	4970	5671	7167	8656	9832	10286
**Henan**	2050	2307	2669	2611	2588	3190	3078	3400	3552	4412	5052
**Hubei**	950	1132	1267	1216	1370	1525	1622	1987	1952	2444	3231
**Hunan**	1615	1577	1723	1651	1761	1988	1995	2386	2401	2691	3223
**Guangdong**	4033	3997	4079	4431	4695	4710	5879	6233	5927	6436	8256
**Guangxi**	1548	1541	1687	1708	1843	1757	1989	2313	2246	2711	2859
**Hainan**	133	138	170	216	239	232	260	313	311	331	446
**Chongqing**	622	792	981	987	1170	1388	1443	1760	1604	1817	2072
**Sichuan**	2178	1707	1844	1914	2068	2314	2433	3142	2781	3276	3655
**Guizhou**	408	503	459	575	642	965	907	1349	1130	1350	1531
**Yunnan**	888	1052	1261	1295	1221	1297	1364	1716	1792	2238	2515
**Tibet**	16	23	27	28	36	35	42	94	71	91	117
**Shaanxi**	716	926	704	800	810	1196	1074	1549	1595	1733	2023
**Gansu**	467	486	525	579	601	725	887	999	1063	1151	1161
**Qinghai**	48	51	67	76	81	114	171	209	217	239	278
**Ningxia**	144	161	207	224	256	285	337	467	511	507	556
**Xinjiang**	414	470	553	611	690	740	748	900	893	939	958

	2007	2008	2009	2010	2011	2012	2013	2014	2015	2016
**China**	95668	97701	108408	118817	131630	132785	139016	142148	130662	129503
**Beijing**	864	707	809	730	772	632	616	476	361	321
**Tianjin**	305	196	205	212	162	163	198	220	154	151
**Hebei**	5478	5041	4940	4677	6128	6264	6219	5173	4243	4447
**Shanxi**	1854	1573	1640	2402	3126	3120	3157	2958	2234	2196
**Inner Mongolia**	1789	1903	2483	3107	3725	3448	3706	3646	3199	3339
**Liaoning**	2353	2521	3237	3158	3839	3428	3439	3283	2483	2107
**Jilin**	1800	1987	2475	2640	2790	2240	2262	2445	2114	1699
**Heilongjiang**	1211	1095	1204	1479	1735	1798	1685	1521	1228	1289
**Shanghai**	190	172	134	57	48	43	41	37	23	21
**Jiangsu**	5915	6130	6827	6226	6493	7209	6799	7272	6490	6265
**Zhejiang**	6168	5745	5737	5818	5936	5602	5925	5829	5127	4743
**Anhui**	7914	8689	9700	10057	10472	11231	11799	12426	12182	12107
**Fujian**	3449	3591	4054	4207	4877	4769	5056	4920	4747	4775
**Jiangxi**	3614	4077	4541	4442	4825	4746	5266	5558	5144	5021
**Shandong**	9773	9157	8429	9108	10398	9804	9088	9162	8134	8327
**Henan**	6461	5571	6427	6123	6652	7196	7965	8135	7543	6850
**Hubei**	3527	3982	4376	5523	5616	5776	5771	5901	5548	5580
**Hunan**	3839	4254	4983	5836	5940	5780	6103	6502	6005	6080
**Guangdong**	8238	7981	7911	8382	8416	6921	7596	8286	7833	7856
**Guangxi**	3464	4423	5479	5674	6741	7084	7527	7337	7329	7647
**Hainan**	451	489	723	778	1013	1246	1400	1498	1493	1445
**Chongqing**	2357	2503	2566	3341	3778	3536	3870	4172	4103	3935
**Sichuan**	4415	4634	5909	8390	9026	7992	7949	8264	7654	7672
**Guizhou**	1629	1655	2113	2931	3978	4701	5566	6452	6439	6759
**Yunnan**	2760	3045	3824	4039	4748	5371	6125	6417	6039	6867
**Tibet**	123	128	171	207	217	266	270	309	407	524
**Shaanxi**	2335	2781	3247	3621	4211	4267	4519	4758	4295	3514
**Gansu**	1182	1285	1377	1798	2199	2476	2967	3271	3043	2864
**Qinghai**	348	366	399	522	751	906	1110	1116	1017	1054
**Ningxia**	686	683	856	1067	1153	1163	1357	1256	1174	1286
**Xinjiang**	1177	1337	1632	2264	1865	3606	3662	3546	2877	2761

Table S4 Clinker-to-cement ratios of China and its provinces (%)

	1996	1997	1998	1999	2000	2001	2002	2003	2004	2005	2006
**China average**	72%	72%	72%	72%	72%	72%	72%	76%	70%	72%	71%
**Beijing**	66%	66%	66%	66%	66%	66%	66%	69%	64%	65%	72%
**Tianjin**	47%	47%	47%	47%	47%	47%	47%	49%	46%	46%	40%
**Hebei**	55%	55%	55%	55%	55%	55%	55%	58%	54%	55%	54%
**Shanxi**	67%	67%	67%	67%	67%	67%	67%	70%	65%	66%	65%
**Inner Mongolia**	72%	72%	72%	72%	72%	72%	72%	76%	70%	71%	64%

**Liaoning**	64%	64%	64%	64%	64%	64%	64%	68%	62%	63%	59%
**Jilin**	91%	91%	91%	91%	91%	91%	91%	96%	89%	90%	88%
**Heilongjiang**	72%	72%	72%	72%	72%	72%	72%	76%	70%	71%	73%
**Shanghai**	34%	34%	34%	34%	34%	34%	34%	36%	33%	34%	17%
**Jiangsu**	58%	58%	58%	58%	58%	58%	58%	61%	56%	57%	59%
**Zhejiang**	73%	73%	73%	73%	73%	73%	73%	76%	71%	72%	71%
**Anhui**	154%	154%	154%	154%	154%	154%	154%	162%	150%	152%	139%
**Fujian**	83%	83%	83%	83%	83%	83%	83%	87%	80%	82%	76%
**Jiangxi**	67%	67%	67%	67%	67%	67%	67%	71%	65%	66%	68%
**Shandong**	69%	69%	69%	69%	69%	69%	69%	73%	67%	68%	62%
**Henan**	69%	69%	69%	69%	69%	69%	69%	72%	67%	68%	66%
**Hubei**	55%	55%	55%	55%	55%	55%	55%	58%	54%	54%	62%
**Hunan**	73%	73%	73%	73%	73%	73%	73%	77%	71%	72%	70%
**Guangdong**	79%	79%	79%	79%	79%	79%	79%	83%	77%	78%	85%
**Guangxi**	83%	83%	83%	83%	83%	83%	83%	87%	81%	82%	78%
**Hainan**	75%	75%	75%	75%	75%	75%	75%	79%	73%	74%	76%
**Chongqing**	83%	83%	83%	83%	83%	83%	83%	87%	80%	82%	79%
**Sichuan**	74%	74%	74%	74%	74%	74%	74%	78%	72%	73%	72%
**Guizhou**	81%	81%	81%	81%	81%	81%	81%	85%	79%	80%	80%
**Yunnan**	80%	80%	80%	80%	80%	80%	80%	84%	78%	79%	76%
**Tibet**	72%	72%	72%	72%	72%	72%	72%	76%	70%	71%	70%
**Shaanxi**	81%	81%	81%	81%	81%	81%	81%	85%	79%	80%	81%
**Gansu**	82%	82%	82%	82%	82%	82%	82%	87%	80%	81%	80%
**Qinghai**	65%	65%	65%	65%	65%	65%	65%	69%	63%	64%	75%
**Ningxia**	90%	90%	90%	90%	90%	90%	90%	95%	88%	89%	78%
**Xinjiang**	76%	76%	76%	76%	76%	76%	76%	80%	74%	75%	78%

	**2007**	**2008**	**2009**	**2010**	**2011**	**2012**	**2013**	**2014**	**2015**	**2016**
**China average**	70%	69%	66%	63%	63%	60%	57%	57%	55%	54%
**Beijing**	74%	80%	75%	70%	85%	73%	69%	69%	69%	69%
**Tianjin**	50%	35%	29%	25%	17%	20%	21%	21%	21%	21%
**Hebei**	58%	54%	45%	37%	43%	49%	49%	49%	49%	49%
**Shanxi**	67%	63%	59%	65%	77%	63%	62%	62%	62%	62%
**Inner Mongolia**	62%	58%	55%	57%	58%	58%	58%	58%	58%	58%
**Liaoning**	60%	61%	67%	66%	67%	63%	57%	57%	57%	57%
**Jilin**	86%	85%	90%	86%	75%	70%	67%	67%	67%	67%
**Heilongjiang**	69%	51%	46%	41%	40%	46%	42%	42%	42%	42%
**Shanghai**	20%	21%	17%	8%	6%	6%	6%	6%	6%	6%
**Jiangsu**	50%	46%	46%	40%	44%	43%	38%	38%	38%	38%
**Zhejiang**	59%	56%	53%	51%	49%	49%	48%	48%	48%	48%
**Anhui**	147%	141%	136%	125%	111%	####	97%	97%	97%	97%
**Fujian**	77%	77%	74%	71%	73%	67%	64%	64%	64%	64%
**Jiangxi**	71%	76%	74%	71%	71%	64%	57%	57%	57%	57%

**Shandong**	65%	65%	58%	61%	70%	65%	56%	56%	56%	56%
**Henan**	69%	54%	54%	53%	49%	49%	48%	48%	48%	48%
**Hubei**	63%	68%	62%	61%	60%	57%	53%	53%	53%	53%
**Hunan**	68%	69%	65%	66%	64%	56%	54%	54%	54%	54%
**Guangdong**	83%	82%	78%	72%	67%	61%	57%	57%	57%	57%
**Guangxi**	80%	84%	84%	75%	78%	72%	69%	69%	69%	69%
**Hainan**	71%	74%	77%	62%	68%	76%	71%	71%	71%	71%
**Chongqing**	79%	77%	70%	72%	77%	65%	63%	63%	63%	63%
**Sichuan**	69%	74%	65%	65%	63%	60%	57%	57%	57%	57%
**Guizhou**	79%	80%	79%	77%	76%	71%	68%	68%	68%	68%
**Yunnan**	77%	74%	76%	70%	71%	68%	68%	68%	68%	68%
**Tibet**	77%	76%	91%	95%	95%	95%	92%	92%	92%	92%
**Shaanxi**	74%	76%	72%	66%	65%	57%	53%	53%	53%	53%
**Gansu**	77%	79%	75%	74%	81%	69%	67%	67%	67%	67%
**Qinghai**	80%	79%	65%	64%	73%	65%	61%	61%	61%	61%
**Ningxia**	81%	76%	80%	75%	80%	73%	71%	71%	71%	71%
**Xinjiang**	78%	79%	80%	92%	60%	85%	71%	71%	71%	71%

Table S5 Coal consumption from the cement industry of China and its provinces (10 thousand tonnes)

	**1996**	**1997**	**1998**	**1999**	**2000**	**2001**	**2002**	**2003**	**2004**	**2005**	**2006**
**China**	6783	7093	7285	7744	8136	9084	9885	11709	13102	14432	16310
**Beijing**	89	95	106	110	114	109	120	135	165	160	157
**Tianjin**	24	24	22	30	31	39	44	52	61	60	63
**Hebei**	322	361	382	402	458	468	556	654	732	737	878
**Shanxi**	141	156	157	158	133	172	185	214	263	253	263
**Inner Mongolia**	91	107	115	128	147	160	164	218	297	375	360
**Liaoning**	257	274	255	260	297	314	323	365	376	401	430
**Jilin**	87	91	94	97	112	132	130	163	194	250	330
**Heilongjiang**	91	100	107	122	129	136	136	157	164	171	189
**Shanghai**	13	10	10	7	9	12	10	21	28	30	32
**Jiangsu**	374	379	371	417	439	493	569	735	832	909	1031
**Zhejiang**	391	384	394	431	484	536	645	805	987	1021	1113
**Anhui**	586	606	518	567	508	621	632	805	913	878	1104
**Fujian**	196	202	198	245	203	233	226	317	317	369	418
**Jiangxi**	123	123	137	157	175	189	232	297	366	435	552
**Shandong**	636	669	636	692	763	835	949	1139	1485	1653	1964
**Henan**	382	429	492	483	475	587	564	592	668	813	888
**Hubei**	331	394	437	421	470	526	557	648	687	843	748
**Hunan**	361	352	381	367	387	439	439	499	542	595	658
**Guangdong**	743	735	744	811	851	858	1066	1074	1102	1173	1295
**Guangxi**	302	300	326	331	354	339	382	422	442	523	583
**Hainan**	22	23	28	36	40	39	43	49	53	55	57
**Chongqing**	119	151	186	188	220	263	272	315	310	344	391

**Sichuan**	444	348	373	388	415	467	488	599	573	661	848
**Guizhou**	74	91	82	103	114	172	161	228	206	241	271
**Yunnan**	211	250	297	306	286	305	319	382	431	527	610
**Tibet**	4	6	7	7	9	9	10	21	18	22	21
**Shaanxi**	129	167	126	144	144	214	191	262	291	310	382
**Gansu**	85	88	94	105	108	130	159	170	195	207	213
**Qinghai**	12	12	16	19	20	28	41	48	54	58	65
**Ningxia**	28	31	40	43	49	54	64	84	100	97	110
**Xinjiang**	116	132	154	171	191	206	207	237	253	261	285

	**2007**	**2008**	**2009**	**2010**	**2011**	**2012**	**2013**	**2014**	**2015**	**2016**
**China**	17433	17607	18662	21601	23888	23012	25414	26122	24932	25587
**Beijing**	155	132	141	137	146	121	125	97	76	70
**Tianjin**	61	38	33	39	33	32	36	40	29	30
**Hebei**	928	776	773	896	1110	960	937	782	666	722
**Shanxi**	266	219	233	306	428	412	417	392	307	312
**Inner Mongolia**	394	348	459	560	636	563	602	594	541	584
**Liaoning**	491	449	450	446	525	551	608	582	457	401
**Jilin**	420	442	604	673	725	581	611	663	595	495
**Heilongjiang**	231	243	246	334	391	375	385	349	292	318
**Shanghai**	33	29	24	20	17	15	14	13	8	8
**Jiangsu**	1029	1118	976	1039	1044	1048	1124	1207	1117	1116
**Zhejiang**	1136	1025	964	1004	1007	953	1033	1020	931	891
**Anhui**	1292	1439	1462	1653	1800	1833	2043	2158	2196	2258
**Fujian**	535	546	560	605	744	715	783	765	766	797
**Jiangxi**	658	743	738	749	807	796	976	1033	992	1002
**Shandong**	1845	1587	1634	1680	1909	1689	1786	1806	1664	1763
**Henan**	774	703	834	818	906	897	1017	1042	1003	942
**Hubei**	756	858	854	1093	1187	1195	1281	1314	1282	1334
**Hunan**	803	797	871	1000	1001	1027	1105	1182	1133	1187
**Guangdong**	1386	1355	1271	1452	1535	1185	1394	1526	1497	1554
**Guangxi**	649	766	909	1051	1299	1359	1494	1461	1515	1636
**Hainan**	66	82	115	154	201	225	269	289	299	299
**Chongqing**	439	505	513	651	699	689	767	829	846	840
**Sichuan**	1012	1166	1450	2053	2103	1802	1878	1959	1883	1953
**Guizhou**	301	291	358	510	649	773	943	1097	1136	1234
**Yunnan**	599	649	713	821	907	1029	1179	1239	1210	1424
**Tibet**	22	25	30	35	39	39	40	46	64	85
**Shaanxi**	420	478	555	675	756	700	793	838	785	664
**Gansu**	232	256	283	376	412	469	572	633	611	595
**Qinghai**	69	76	88	117	136	170	223	225	213	228
**Ningxia**	123	126	154	205	232	192	231	214	208	236
**Xinjiang**	309	338	367	445	504	618	748	727	612	608

Table S6 Electricity consumption from the cement industry of China and its provinces (10 thousand 2h)

	1996	1997	1998	1999	2000	2001	2002	2003	2004	2005	2006
**China**	5339	5572	5718	6099	6423	7169	7854	9330	10544	11548	11811
**Beijing**	107	114	128	133	137	132	145	163	199	193	192
**Tianjin**	23	23	21	29	31	38	43	51	59	58	71
**Hebei**	230	258	272	287	326	333	396	466	522	525	687
**Shanxi**	183	202	203	205	172	223	239	277	341	328	334
**Inner Mongolia**	24	28	30	34	39	42	43	57	78	99	8
**Liaoning**	175	187	174	177	203	214	220	249	256	273	281
**Jilin**	84	88	90	93	108	126	124	156	185	239	123
**Heilongjiang**	95	104	112	126	135	141	141	163	171	178	222
**Shanghai**	33	25	25	19	24	32	26	56	73	78	86
**Jiangsu**	371	376	368	413	435	488	564	729	824	901	995
**Zhejiang**	317	312	319	349	392	434	523	653	800	827	861
**Anhui**	341	353	302	330	296	362	368	469	532	511	612
**Fujian**	149	153	150	186	154	177	171	241	241	280	392
**Jiangxi**	100	100	111	127	142	153	188	240	296	352	411
**Shandong**	736	775	736	801	884	967	1099	1319	1720	1914	1373
**Henan**	249	280	322	316	310	384	369	387	436	531	511
**Hubei**	188	224	248	239	267	299	316	368	390	479	439
**Hunan**	242	236	255	246	260	294	294	334	363	399	604
**Guangdong**	578	572	579	631	662	667	829	835	857	912	1018
**Guangxi**	157	156	169	172	184	176	198	219	230	272	269
**Hainan**	39	40	49	62	68	67	75	85	91	95	53
**Chongqing**	125	159	195	197	231	275	285	330	325	360	372
**Sichuan**	327	256	275	286	306	344	360	442	422	487	488
**Guizhou**	52	64	58	73	80	121	113	160	145	170	265
**Yunnan**	152	180	214	220	206	219	230	275	310	379	328
**Tibet**	2	3	3	3	4	4	5	10	8	10	34
**Shaanxi**	87	112	85	96	97	143	128	176	195	208	296
**Gansu**	57	59	64	70	72	88	107	114	131	139	176
**Qinghai**	12	13	17	20	21	29	44	51	57	62	49
**Ningxia**	39	44	56	60	68	76	90	118	140	136	89
**Xinjiang**	67	76	89	99	110	119	120	137	147	151	171

	2007	2008	2009	2010	2011	2012	2013	2014	2015	2016
**China**	14302	14213	16418	18776	21576	22918	25163	25751	24414	24870
**Beijing**	176	193	170	165	159	166	171	132	104	96
**Tianjin**	74	46	50	60	59	66	75	83	60	61
**Hebei**	725	644	807	936	1460	1492	1457	1216	1035	1123
**Shanxi**	338	233	300	394	471	546	552	519	406	413
**Inner Mongolia**	186	390	317	387	582	579	619	611	556	601

**Liaoning**	488	461	489	485	556	566	624	598	469	412
**Jilin**	317	276	352	392	517	589	620	672	603	502
**Heilongjiang**	233	231	301	409	439	397	408	369	309	336
**Shanghai**	73	57	51	44	44	41	39	35	22	21
**Jiangsu**	1112	1149	1028	1095	1078	1061	1138	1222	1131	1130
**Zhejiang**	1084	1194	1257	1309	1292	1215	1318	1301	1187	1137
**Anhui**	706	522	1113	1258	1468	1613	1798	1900	1933	1988
**Fujian**	559	553	573	618	720	716	785	766	767	798
**Jiangxi**	506	494	704	715	902	1023	1255	1329	1276	1289
**Shandong**	1483	1580	1528	1571	2057	1873	1980	2003	1845	1955
**Henan**	750	730	995	977	1255	1202	1363	1397	1344	1263
**Hubei**	452	503	737	943	1053	1212	1298	1332	1300	1353
**Hunan**	728	727	905	1039	1184	1369	1474	1575	1510	1582
**Guangdong**	1134	1083	963	1101	1068	895	1052	1152	1130	1173
**Guangxi**	304	277	380	440	537	740	813	795	824	890
**Hainan**	54	44	81	110	122	136	163	175	181	181
**Chongqing**	401	580	656	832	861	1110	1235	1336	1363	1353
**Sichuan**	970	794	912	1290	1480	1351	1408	1468	1411	1464
**Guizhou**	239	250	274	391	483	801	977	1137	1177	1279
**Yunnan**	229	189	354	408	218	357	409	430	420	494
**Tibet**	31	31	27	32	35	41	43	49	67	89
**Shaanxi**	364	427	418	509	570	647	733	774	725	614
**Gansu**	179	169	205	273	316	373	455	504	486	473
**Qinghai**	57	41	62	82	90	69	91	92	87	93
**Ningxia**	164	142	155	205	178	183	220	204	198	224
**Xinjiang**	187	203	252	305	325	490	593	576	485	482

Table S7 Socioeconomic indexes for IDA

	Clinker export	Proportion of new buildings' types						Fixed capital formation (at 2002 constant price)
		Resdiential building %	Manufacturing building %	Infrastructural building %	Commerial building %	science-education-culture-health building %	Others %	
**Unit**	10^4 tonnes	%	%	%	%	%	%	100 million yuan
**2002**	377	37%	9%	27%	5%	10%	12%	43,797
**2003**	415	35%	11%	24%	4%	11%	15%	52,803
**2004**	526	33%	12%	24%	3%	10%	16%	60,849
**2005**	1,892	34%	14%	24%	3%	10%	15%	69,138
**2006**	3,043	35%	14%	24%	3%	9%	15%	78,371
**2007**	2,849	35%	14%	24%	3%	9%	15%	90,848
**2008**	2,191	36%	13%	25%	3%	8%	15%	101,648
**2009**	1,271	36%	12%	28%	3%	8%	14%	127,525
**2010**	1,255	37%	12%	27%	3%	8%	14%	145,943
**2011**	734	39%	12%	24%	3%	7%	14%	161,841
**2012**	830	42%	12%	23%	4%	7%	13%	178,247
**2013**	985	43%	11%	23%	4%	7%	12%	196,839
**2014**	953	44%	11%	23%	4%	7%	12%	209,688
**2015**	1,165	43%	11%	23%	4%	7%	12%	221,961
**2016**	1,401	43%	10%	24%	5%	7%	11%	235,581

Table S8 Process-related CO_2 emissions from the cement industry of China and its provinces (10 thousand tonnes)

	1996	1997	1998	1999	2000	2001	2002	2003	2004	2005	2006
**China**	17686	18428	18942	20164	21319	23757	26068	32615	33972	37961	43350
**Beijing**	209	224	251	261	272	260	287	340	385	381	437
**Tianjin**	47	48	43	60	63	78	88	110	119	120	120
**Hebei**	901	1012	1079	1131	1300	1323	1579	1954	2027	2083	2295
**Shanxi**	417	460	467	471	398	514	554	674	768	755	865
**Inner Mongolia**	138	164	177	197	228	247	254	355	448	578	698
**Liaoning**	536	572	537	545	630	663	683	814	776	845	958
**Jilin**	266	278	288	298	347	406	402	530	584	769	786
**Heilongjiang**	227	249	270	304	327	342	343	418	405	430	538
**Shanghai**	73	57	57	43	54	73	60	133	161	176	93
**Jiangsu**	1116	1132	1117	1251	1330	1485	1724	2343	2457	2739	3215
**Zhejiang**	1234	1214	1255	1366	1551	1709	2068	2715	3085	3254	3518
**Anhui**	1673	1732	1492	1627	1471	1792	1833	2456	2580	2532	3157
**Fujian**	596	614	607	749	628	716	697	1032	954	1134	1287
**Jiangxi**	342	343	382	437	493	530	654	880	1005	1219	1448
**Shandong**	1857	1958	1875	2033	2264	2467	2815	3558	4297	4881	5106
**Henan**	1018	1145	1325	1296	1285	1583	1528	1688	1763	2190	2508
**Hubei**	472	562	629	604	680	757	805	986	969	1213	1604
**Hunan**	801	783	855	820	874	987	990	1185	1192	1336	1600
**Guangdong**	2002	1984	2025	2199	2330	2338	2918	3094	2942	3195	4098
**Guangxi**	768	765	837	848	915	872	987	1148	1115	1346	1419
**Hainan**	66	69	84	107	119	115	129	155	155	164	221
**Chongqing**	309	393	487	490	581	689	716	874	796	902	1029
**Sichuan**	1081	847	916	950	1026	1149	1208	1560	1380	1626	1814
**Guizhou**	203	250	228	285	319	479	450	670	561	670	760
**Yunnan**	441	522	626	643	606	644	677	852	890	1111	1249
**Tibet**	8	11	13	14	18	18	21	47	35	45	58
**Shaanxi**	355	460	350	397	402	594	533	769	792	860	1004
**Gansu**	232	241	260	287	298	360	441	496	528	571	576
**Qinghai**	24	25	33	38	40	56	85	104	108	119	138
**Ningxia**	71	80	103	111	127	141	167	232	254	252	276
**Xinjiang**	206	233	275	303	342	368	371	447	443	466	476

	2007	2008	2009	2010	2011	2012	2013	2014	2015	2016
**China**	47490	48499	53814	58981	65341	65914	69008	70562	64861	64285
**Beijing**	429	351	402	362	383	314	306	236	179	160
**Tianjin**	151	97	102	105	80	81	98	109	77	75
**Hebei**	2719	2502	2452	2322	3042	3109	3087	2568	2106	2208
**Shanxi**	920	781	814	1192	1552	1549	1567	1468	1109	1090
**Inner Mongolia**	888	945	1233	1542	1849	1712	1840	1810	1588	1658
**Liaoning**	1168	1251	1607	1568	1906	1702	1707	1630	1232	1046

**Jilin**	894	986	1229	1311	1385	1112	1123	1214	1050	843
**Heilongjiang**	601	544	598	734	861	893	837	755	610	640
**Shanghai**	94	85	67	28	24	21	20	18	11	10
**Jiangsu**	2936	3043	3389	3091	3223	3579	3375	3610	3222	3110
**Zhejiang**	3062	2852	2848	2888	2947	2781	2941	2893	2545	2355
**Anhui**	3928	4313	4815	4992	5198	5575	5857	6168	6047	6010
**Fujian**	1712	1783	2012	2088	2421	2367	2510	2443	2356	2370
**Jiangxi**	1794	2024	2254	2205	2395	2356	2614	2759	2553	2492
**Shandong**	4851	4546	4184	4521	5162	4867	4511	4548	4038	4134
**Henan**	3207	2765	3190	3039	3302	3572	3954	4038	3745	3400
**Hubei**	1751	1977	2172	2742	2788	2867	2865	2929	2754	2770
**Hunan**	1906	2112	2474	2897	2949	2869	3030	3227	2981	3018
**Guangdong**	4089	3962	3927	4161	4178	3435	3771	4113	3888	3900
**Guangxi**	1720	2196	2720	2817	3346	3516	3736	3642	3638	3796
**Hainan**	224	243	359	386	503	618	695	744	741	717
**Chongqing**	1170	1242	1274	1658	1875	1756	1921	2071	2037	1953
**Sichuan**	2192	2300	2933	4165	4480	3967	3946	4102	3800	3808
**Guizhou**	809	822	1049	1455	1975	2334	2763	3203	3197	3355
**Yunnan**	1370	1512	1898	2005	2357	2666	3040	3185	2998	3409
**Tibet**	61	64	85	103	108	132	134	153	202	260
**Shaanxi**	1159	1380	1612	1797	2090	2118	2243	2362	2132	1744
**Gansu**	587	638	684	893	1092	1229	1473	1624	1511	1422
**Qinghai**	173	182	198	259	373	450	551	554	505	523
**Ningxia**	341	339	425	530	573	578	673	623	583	639
**Xinjiang**	584	664	810	1124	926	1790	1818	1760	1428	1371

Table S9 Coal-related CO_2 emissions from the cement industry of China and its provinces (10 thousand tonnes)

	1996	1997	1998	1999	2000	2001	2002	2003	2004	2005	2006
**China**	3385	3539	3635	3864	4060	4533	4933	5843	6538	7202	8139
**Beijing**	44	47	53	55	57	55	60	67	82	80	78
**Tianjin**	12	12	11	15	16	20	22	26	30	30	31
**Hebei**	161	180	191	201	228	233	277	326	365	368	438
**Shanxi**	70	78	78	79	66	86	92	107	131	126	131
**Inner Mongolia**	45	54	57	64	73	80	82	109	148	187	180
**Liaoning**	128	137	127	130	148	157	161	182	187	200	215
**Jilin**	44	46	47	49	56	66	65	81	97	125	165
**Heilongjiang**	45	50	54	61	65	68	68	78	82	85	94
**Shanghai**	6	5	5	4	5	6	5	11	14	15	16
**Jiangsu**	187	189	185	208	219	246	284	367	415	454	515
**Zhejiang**	195	192	197	215	242	267	322	402	493	509	555
**Anhui**	292	303	258	283	253	310	316	402	456	438	551
**Fujian**	98	101	99	122	102	116	113	158	158	184	209
**Jiangxi**	61	62	68	78	87	94	116	148	183	217	275

**Shandong**	317	334	317	345	381	417	473	568	741	825	980
**Henan**	190	214	246	241	237	293	282	296	333	406	443
**Hubei**	165	197	218	210	235	262	278	323	343	421	373
**Hunan**	180	176	190	183	193	219	219	249	270	297	329
**Guangdong**	371	367	371	405	425	428	532	536	550	585	646
**Guangxi**	151	150	163	165	177	169	190	211	221	261	291
**Hainan**	11	12	14	18	20	19	21	24	26	27	29
**Chongqing**	59	76	93	94	110	131	136	157	155	172	195
**Sichuan**	222	173	186	194	207	233	244	299	286	330	423
**Guizhou**	37	45	41	51	57	86	80	114	103	120	135
**Yunnan**	105	125	148	153	143	152	159	191	215	263	305
**Tibet**	2	3	3	3	4	4	5	11	9	11	11
**Shaanxi**	65	83	63	72	72	107	95	131	145	155	191
**Gansu**	42	44	47	52	54	65	79	85	97	103	106
**Qinghai**	6	6	8	9	10	14	21	24	27	29	32
**Ningxia**	14	15	20	21	24	27	32	42	50	48	55
**Xinjiang**	58	66	77	85	95	103	103	118	126	130	142

	**2007**	**2008**	**2009**	**2010**	**2011**	**2012**	**2013**	**2014**	**2015**	**2016**
**China**	8699	8786	9312	10779	11920	11483	12681	13035	12441	12768
**Beijing**	77	66	71	69	73	61	62	48	38	35
**Tianjin**	30	19	16	19	17	16	18	20	15	15
**Hebei**	463	387	386	447	554	479	468	390	332	360
**Shanxi**	133	109	116	153	213	206	208	195	153	156
**Inner Mongolia**	197	174	229	280	318	281	300	296	270	292
**Liaoning**	245	224	224	222	262	275	303	291	228	200
**Jilin**	210	220	302	336	362	290	305	331	297	247
**Heilongjiang**	115	121	123	167	195	187	192	174	146	158
**Shanghai**	16	15	12	10	8	8	7	6	4	4
**Jiangsu**	513	558	487	519	521	523	561	602	558	557
**Zhejiang**	567	511	481	501	502	475	516	509	465	445
**Anhui**	645	718	730	825	898	914	1019	1077	1096	1127
**Fujian**	267	273	280	302	372	357	391	382	382	398
**Jiangxi**	329	371	368	374	403	397	487	516	495	500
**Shandong**	921	792	815	838	952	843	891	901	830	880
**Henan**	386	351	416	408	452	447	507	520	500	470
**Hubei**	377	428	426	545	592	597	639	656	640	666
**Hunan**	401	398	435	499	499	512	552	590	565	592
**Guangdong**	692	676	634	725	766	591	696	761	747	775
**Guangxi**	324	382	454	525	648	678	746	729	756	816
**Hainan**	33	41	57	77	100	112	134	144	149	149
**Chongqing**	219	252	256	325	349	344	383	414	422	419
**Sichuan**	505	582	724	1025	1049	899	937	977	939	974

**Guizhou**	150	145	179	255	324	386	471	547	567	616
**Yunnan**	299	324	356	410	453	514	588	618	604	710
**Tibet**	11	12	15	17	19	19	20	23	32	42
**Shaanxi**	209	238	277	337	377	349	396	418	392	332
**Gansu**	116	128	141	188	205	234	285	316	305	297
**Qinghai**	34	38	44	58	68	85	111	112	106	114
**Ningxia**	61	63	77	102	116	96	115	107	104	118
**Xinjiang**	154	169	183	222	252	308	373	363	305	303

Table S10 Electricity-related CO_2 emissions from the cement industry of China and its provinces (10thousand tonnes)

	**1996**	**1997**	**1998**	**1999**	**2000**	**2001**	**2002**	**2003**	**2004**	**2005**	**2006**
**China**	5339	5572	5718	6099	6423	7169	7854	9330	10544	11548	11811
**Beijing**	107	114	128	133	137	132	145	163	199	193	192
**Tianjin**	23	23	21	29	31	38	43	51	59	58	71
**Hebei**	230	258	272	287	326	333	396	466	522	525	687
**Shanxi**	183	202	203	205	172	223	239	277	341	328	334
**Inner Mongolia**	24	28	30	34	39	42	43	57	78	99	8
**Liaoning**	175	187	174	177	203	214	220	249	256	273	281
**Jilin**	84	88	90	93	108	126	124	156	185	239	123
**Heilongjiang**	95	104	112	126	135	141	141	163	171	178	222
**Shanghai**	33	25	25	19	24	32	26	56	73	78	86
**Jiangsu**	371	376	368	413	435	488	564	729	824	901	995
**Zhejiang**	317	312	319	349	392	434	523	653	800	827	861
**Anhui**	341	353	302	330	296	362	368	469	532	511	612
**Fujian**	149	153	150	186	154	177	171	241	241	280	392
**Jiangxi**	100	100	111	127	142	153	188	240	296	352	411
**Shandong**	736	775	736	801	884	967	1099	1319	1720	1914	1373
**Henan**	249	280	322	316	310	384	369	387	436	531	511
**Hubei**	188	224	248	239	267	299	316	368	390	479	439
**Hunan**	242	236	255	246	260	294	294	334	363	399	604
**Guangdong**	578	572	579	631	662	667	829	835	857	912	1018
**Guangxi**	157	156	169	172	184	176	198	219	230	272	269
**Hainan**	39	40	49	62	68	67	75	85	91	95	53
**Chongqing**	125	159	195	197	231	275	285	330	325	360	372
**Sichuan**	327	256	275	286	306	344	360	442	422	487	488
**Guizhou**	52	64	58	73	80	121	113	160	145	170	265
**Yunnan**	152	180	214	220	206	219	230	275	310	379	328
**Tibet**	2	3	3	3	4	4	5	10	8	10	34
**Shaanxi**	87	112	85	96	97	143	128	176	195	208	296
**Gansu**	57	59	64	70	72	88	107	114	131	139	176
**Qinghai**	12	13	17	20	21	29	44	51	57	62	49
**Ningxia**	39	44	56	60	68	76	90	118	140	136	89
**Xinjiang**	67	76	89	99	110	119	120	137	147	151	171

	2007	2008	2009	2010	2011	2012	2013	2014	2015	2016
**China**	14302	14213	16418	18776	21576	22918	25163	25751	24414	24870
**Beijing**	176	193	170	165	159	166	171	132	104	96
**Tianjin**	74	46	50	60	59	66	75	83	60	61
**Hebei**	725	644	807	936	1460	1492	1457	1216	1035	1123
**Shanxi**	338	233	300	394	471	546	552	519	406	413
**Inner Mongolia**	186	390	317	387	582	579	619	611	556	601
**Liaoning**	488	461	489	485	556	566	624	598	469	412
**Jilin**	317	276	352	392	517	589	620	672	603	502
**Heilongjiang**	233	231	301	409	439	397	408	369	309	336
**Shanghai**	73	57	51	44	44	41	39	35	22	21
**Jiangsu**	1112	1149	1028	1095	1078	1061	1138	1222	1131	1130
**Zhejiang**	1084	1194	1257	1309	1292	1215	1318	1301	1187	1137
**Anhui**	706	522	1113	1258	1468	1613	1798	1900	1933	1988
**Fujian**	559	553	573	618	720	716	785	766	767	798
**Jiangxi**	506	494	704	715	902	1023	1255	1329	1276	1289
**Shandong**	1483	1580	1528	1571	2057	1873	1980	2003	1845	1955
**Henan**	750	730	995	977	1255	1202	1363	1397	1344	1263
**Hubei**	452	503	737	943	1053	1212	1298	1332	1300	1353
**Hunan**	728	727	905	1039	1184	1369	1474	1575	1510	1582
**Guangdong**	1134	1083	963	1101	1068	895	1052	1152	1130	1173
**Guangxi**	304	277	380	440	537	740	813	795	824	890
**Hainan**	54	44	81	110	122	136	163	175	181	181
**Chongqing**	401	580	656	832	861	1110	1235	1336	1363	1353
**Sichuan**	970	794	912	1290	1480	1351	1408	1468	1411	1464
**Guizhou**	239	250	274	391	483	801	977	1137	1177	1279
**Yunnan**	229	189	354	408	218	357	409	430	420	494
**Tibet**	31	31	27	32	35	41	43	49	67	89
**Shaanxi**	364	427	418	509	570	647	733	774	725	614
**Gansu**	179	169	205	273	316	373	455	504	486	473
**Qinghai**	57	41	62	82	90	69	91	92	87	93
**Ningxia**	164	142	155	205	178	183	220	204	198	224
**Xinjiang**	187	203	252	305	325	490	593	576	485	482

Table S11 Total CO_2 emissions from the cement industry of China and its provinces (10 thousand tonnes)

	1996	1997	1998	1999	2000	2001	2002	2003	2004	2005	2006
**China**	26410	27539	28295	30127	31802	35460	38855	47788	51054	56710	63299
**Beijing**	360	386	432	448	466	447	492	570	667	654	706
**Tianjin**	82	83	75	104	109	136	152	187	208	208	222
**Hebei**	1292	1450	1542	1619	1855	1890	2253	2746	2914	2976	3420
**Shanxi**	670	740	748	755	637	823	886	1057	1240	1209	1330
**Inner Mongolia**	207	245	264	294	340	370	380	521	674	864	886

**Liaoning**	840	896	838	851	981	1034	1064	1245	1219	1318	1453
**Jilin**	393	412	424	439	510	598	591	767	866	1133	1074
**Heilongjiang**	367	404	435	491	526	551	551	659	658	693	854
**Shanghai**	112	87	87	65	82	112	91	199	248	269	196
**Jiangsu**	1673	1697	1671	1872	1984	2219	2573	3439	3696	4093	4725
**Zhejiang**	1746	1717	1771	1930	2185	2411	2913	3769	4378	4591	4934
**Anhui**	2307	2388	2052	2240	2020	2464	2517	3327	3568	3482	4320
**Fujian**	843	867	855	1057	884	1009	981	1431	1353	1598	1888
**Jiangxi**	503	504	561	643	721	778	958	1269	1484	1788	2134
**Shandong**	2910	3068	2929	3179	3528	3851	4387	5445	6757	7620	7459
**Henan**	1457	1640	1892	1853	1832	2260	2178	2370	2533	3127	3462
**Hubei**	825	983	1096	1053	1182	1318	1400	1678	1702	2113	2416
**Hunan**	1223	1194	1301	1248	1327	1500	1503	1768	1825	2031	2532
**Guangdong**	2950	2923	2975	3235	3417	3434	4279	4466	4349	4693	5763
**Guangxi**	1076	1071	1169	1185	1275	1217	1376	1578	1565	1879	1979
**Hainan**	116	121	147	188	207	201	225	265	272	287	303
**Chongqing**	493	627	775	780	922	1095	1136	1361	1275	1434	1596
**Sichuan**	1630	1277	1376	1429	1539	1725	1811	2301	2088	2443	2726
**Guizhou**	291	358	327	409	456	686	644	944	808	960	1160
**Yunnan**	698	827	988	1016	954	1016	1066	1317	1414	1753	1881
**Tibet**	12	17	20	21	26	26	31	68	52	67	103
**Shaanxi**	507	656	497	566	570	844	757	1075	1132	1223	1492
**Gansu**	332	345	371	410	425	513	627	695	757	814	859
**Qinghai**	42	44	59	67	71	99	150	179	191	209	219
**Ningxia**	124	139	178	193	219	245	290	392	443	436	420
**Xinjiang**	331	375	440	487	548	589	594	702	716	747	789

	**2007**	**2008**	**2009**	**2010**	**2011**	**2012**	**2013**	**2014**	**2015**	**2016**
**China**	70491	71497	79544	88535	98838	100316	106852	109349	101716	101923
**Beijing**	682	610	642	596	615	540	539	417	321	290
**Tianjin**	256	162	168	184	156	163	191	213	152	151
**Hebei**	3908	3534	3645	3705	5055	5080	5012	4175	3474	3691
**Shanxi**	1391	1123	1230	1739	2236	2300	2327	2183	1668	1659
**Inner Mongolia**	1271	1508	1778	2209	2749	2572	2759	2717	2414	2550
**Liaoning**	1901	1937	2321	2275	2723	2543	2634	2518	1929	1658
**Jilin**	1420	1483	1882	2039	2263	1991	2048	2217	1950	1592
**Heilongjiang**	949	896	1021	1310	1495	1476	1437	1298	1065	1134
**Shanghai**	183	157	130	82	76	70	66	60	38	36
**Jiangsu**	4562	4750	4904	4704	4822	5162	5074	5434	4911	4797
**Zhejiang**	4713	4557	4585	4698	4741	4471	4775	4703	4197	3936
**Anhui**	5279	5554	6658	7075	7564	8103	8675	9145	9076	9125
**Fujian**	2538	2608	2864	3008	3512	3440	3685	3590	3505	3566
**Jiangxi**	2628	2889	3326	3294	3699	3776	4356	4603	4325	4281

**Shandong**	7255	6918	6528	6931	8171	7583	7383	7453	6713	6968
**Henan**	4343	3847	4602	4425	5009	5221	5824	5955	5589	5133
**Hubei**	2580	2907	3336	4230	4433	4675	4802	4917	4693	4788
**Hunan**	3034	3237	3814	4435	4632	4751	5055	5392	5056	5193
**Guangdong**	5916	5721	5524	5986	6012	4921	5519	6027	5765	5847
**Guangxi**	2347	2854	3554	3781	4532	4934	5295	5167	5218	5503
**Hainan**	311	328	498	573	725	867	992	1063	1071	1048
**Chongqing**	1790	2074	2185	2815	3086	3210	3539	3820	3822	3726
**Sichuan**	3667	3676	4569	6480	7010	6217	6291	6548	6150	6246
**Guizhou**	1198	1216	1502	2100	2782	3520	4211	4887	4941	5250
**Yunnan**	1898	2025	2608	2823	3028	3537	4038	4233	4021	4613
**Tibet**	103	107	127	152	162	193	197	225	301	391
**Shaanxi**	1732	2046	2307	2644	3038	3114	3372	3554	3249	2690
**Gansu**	881	934	1030	1353	1613	1836	2213	2443	2302	2192
**Qinghai**	264	261	304	399	530	604	753	758	698	730
**Ningxia**	566	544	657	837	867	856	1008	934	884	981
**Xinjiang**	926	1035	1245	1651	1502	2588	2783	2698	2218	2156

Reference

Ang, B. W. 2004. Decomposition analysis for policymaking in energy. *Energy Policy* 32(9): 1131-1139.

Ang, B. W. 2005. The LMDI approach to decomposition analysis: a practical guide. *Energy Policy* 33(7): 867-871.
